# Metabolic reprogramming and immune regulation in acute myeloid leukemia

**DOI:** 10.3389/fimmu.2025.1655005

**Published:** 2025-12-10

**Authors:** Xiaogang Hao, Chenyang Fan, Lixiang Yan, Reaila Jianati, Yifei Guo, Xinli Zhou, Gengda Zhu, Yucheng Zhang, Chengyulong Zheng, Ying Zhang, Zhexin Shi

**Affiliations:** First Teaching Hospital of Tianjin University of Traditional Chinese Medicine, National Clinical Research Center for Chinese Medicine Acupuncture and Moxibustion, Tianjin, China

**Keywords:** AML, metabolic reprogramming, immune regulation, review, metabolism

## Abstract

The most prevalent kind of acute leukemia in adults is acute myeloid leukemia (AML). While some individuals have had better effectiveness due to advancements in targeted medications, recurrence after remission and inadequate treatment specificity continue to be significant therapeutic problems. By controlling essential metabolic pathways and metabolites, metabolic reprogramming, a crucial strategy for cellular adaptability to energy needs, modifies cellular metabolic rhythms. In addition to being involved in immune cell proliferation, differentiation, and effector function, this pathway is also essential for leukemogenesis and survival signaling in AML. By altering the expression of immune molecules, the release of certain metabolites (such as lactate, ROS, glutamine, etc.) has a significant impact on the immune response to tumors. It is noteworthy that the metabolic interactions between immune cells and AML cells form a distinct pattern of energy competition in the tumor microenvironment. This study examined the new approach of targeting metabolic pathways to improve immunotherapy, systematically clarified the regulatory mechanism of metabolic reprogramming between AML cells and immune cells to counteract tumor immunity, and concentrated on the synergistic effect of current therapies and metabolic interventions. These findings offered a fresh perspective on how to fully realize the potential of metabolic therapy for AML.

## 1 Introduction

The most prevalent kind of acute leukemia in adults, accounting for almost 80% of cases, is acute myeloid leukemia (AML), a heterogeneous hematologic malignancy that arises from the clonal proliferation of primitive cells and has a 31.7% 5-year overall survival rate (1). Previous researchers have believed that an important reason for the extremely poor prognosis of AML is the metabolic abnormality of AML cells. Metabolic reprogramming may lead to the progression of the disease to acute leukemia clones and adaptation in the bone marrow. Moreover, Leukemia stem cells (LSCs) can escape from treatment, leading to recurrence (2, 3). Although hematopoietic stem cell transplantation and chemotherapy are currently the cornerstones of AML treatment, recurrence following these treatments can still significantly affect the prognosis of the patient. The process by which a cell adapts its metabolic program to satisfy its energy requirements in various contexts is known as metabolic reprogramming (MR). This process can assist the tumor cell to protect itself from harmful external stimuli and can also offer it new roles. The metabolism of glucose, lipids, amino acids, and other substances are all part of metabolic reprogramming.

The reprogramming of immune cell metabolism is closely linked to the malignant transformation of tumor cells. For instance, immune cells that have shifted from oxidative phosphorylation to glycolysis provide energy for cell proliferation and effector functions in the form of ATP and biosynthetic precursors (4, 5). In addition to competing with immune cells for essential nutrients (6). AML cells’ metabolic reprogramming produces abnormal metabolites or intermediates that are crucial in controlling the growth, differentiation, activation, and function of immune cells.

The metabolism of AML cells interacts with that of immune cells, which in turn influences immunological actions. Notably, every subpopulation of immune cells has a unique metabolic program that, when combined with the signaling pathways of the cell, can help these cells overcome the adverse effects of metabolic constraint (7). Therefore, the primary focus should be on metabolic reprogramming of AML cells, as this can selectively alter the immune response (8). The revelation of tumors for etiology and metabolic targets has opened up new avenues for multifaceted AML treatment and relapse prevention.

The metabolic connections between AML and immunity, the suppression of immunity by AML cells, the metabolic counteraction of AML by immune cells, and the current treatment research will all be included in this review. Together, we will present an overview of the latest developments, work to improve the effectiveness of AML immunotherapy and the anti-tumor immune response, and provide fresh concepts for the advancement of AML treatment in the future.

## 2 Metabolic pattern analysis

### 2.1 Metabolic characteristics of acute myeloid leukemia cells

AML is caused by a variety of biological mechanisms. Hematopoietic stem cells (HSPC) can develop into LSCs with abnormal differentiation traits when they undergo malignant transformation due to gene abnormalities. These LSCs undergo metabolic reprogramming to develop distinct energy-dependent traits (9). By competitively occupying ecological niches in the bone marrow microenvironment (BMM), LSCs create malignant clonal populations. Their secreted inhibitory factors (CCL3 Block normal hematopoietic differentiation and reshape pro-leukemia niches) (10) can disrupt normal hematopoietic processes and take over the pro-proliferative signaling pathway, which can result in unchecked AML cell proliferation and ultimately bone marrow hematopoietic failure (11).

The rapid proliferation of tumor cells requires a large amount of energy and metabolic precursors (12). During the course of disease development, due to genomic instability, the progeny cells of the founding clone will continuously acquire new genetic or epigenetic variations, thereby evolving into multiple “subclones”. As hematological malignancies, the subclonal heterogeneity of AML is also profoundly reflected in the cellular metabolic phenotype (13). In AML, this reprogramming is not uniform but shows significant heterogeneity, mainly reflected in the differences in dependence on the two major energy generation pathways of oxidative phosphorylation (OXPHOS) and glycolysis. LSCs are particularly dependent on oxidative phosphorylation (OXPHOS) for energy supply. In the hypoxic BMM, LSCs construct a malignant ecological niche conducive to their survival and proliferation by regulating fatty acid and amino acid metabolism (6). Notably, LSCs proliferation requires high levels of fatty acid oxidation (FAO) with relatively low fatty acid synthesis (FAS) activity (14).

To meet the need for ATP for quick multiplication, AML cells preferentially use the glycolytic pathway for energy, a characteristic of metabolic reprogramming in tumor cells known as the Warburg effect (15). AML cells effectively produce ATP and other essential metabolic intermediates in addition to glycolysis by controlling the metabolism of fatty acids, amino acids, and other substances. The synthesis of NADPH, which is necessary to fend off oxidative stress and encourage cell division, and fatty acid oxidation (FAO) are noteworthy sources of energy for AML cells (16).

Because of their exceptional metabolic flexibility, AML cells can adapt their metabolism to changing dietary conditions and stress levels, allowing them to constantly produce ATP and biomolecules. For instance, by causing insulin resistance and preventing insulin production, LSCs may preferentially take up glucose to sustain malignant development (17, 18). Furthermore, AML cells have the ability to rewire nutrition-sensing pathways so that they continue to function metabolically during food deprivation (19).

In conclusion, AML cells have extremely dynamic metabolic control, and they can choose the best metabolic pattern in response to changes in their surroundings to maintain their advantage in survival.

### 2.2 Metabolic characteristics of immune cells

Natural killer cells, neutrophils, monocytes, eosinophils, basophils, macrophages, and lymphocytes are the primary immune cells linked to AML. The notable differential in energy metabolism between resting and active immune cells is a fundamental aspect of immune system metabolism. It is significant because these extremely diverse metabolic patterns are also found in immune cells (20), and the intricate metabolic profile of AML cells has already been described.

T lymphocytes are essential for eliminating infections and destroying AML cells. When stimulated to become effector T cells, their metabolic pattern changes dramatically. While resting memory T cells primarily rely on OXPHOS and FAO metabolism for ATP production, they can exhibit 20–50 times higher levels of glycolysis and a marked increase in fatty acid uptake (21, 22). Different immune cells have distinct metabolic profiles: M2-like macrophages use both FAO and OXPHOS, T helper 17 (Th17) cells and regulatory T cells (Tregs) are more prone to FAO metabolism, while activated neutrophils, M1-type macrophages, and dendritic cells (DCs) are primarily glycolytic (23). These metabolic patterns dictate the differentiation fate of immune cells in addition to promoting cellular proliferation. Understanding the nature of immune responses and regulatory mechanisms hence requires a thorough investigation of immune cell metabolic reprogramming.

### 2.3 Mechanisms of trophic competition between AML cells and immune cells

LSCs in the bone marrow niche that have undergone aberrant differentiation give rise to AML cells. A distinct survival advantage for LSCs is offered by the BMM, a dynamic and intricate system made up of both cellular and non-cellular components. According to studies, LSCs utilize and regulate the specific TME of AML to facilitate their own development during initiation, growth and metastasis (24).

AML cells undergo metabolic reprogramming during transformation and proliferation that is very similar to activated rapidly proliferating immune cells. In the Tumor microenvironment (TME) of AML, AML cells have been demonstrated to be the primary consumers of glucose. Due to the metabolic rate of aerobic glycolysis being much faster than mitochondrial respiration, this metabolic competition inhibits the function of immune cells, which is conducive to the survival and proliferation of AML cells (25). Furthermore, a hypoxic, acidic, and low-nutrient TME is created by the massive amount of lactic acid generated by glycolysis, further impairing immune cell activity (26). AML cells competitively absorb glutamine, a crucial ingredient for cell development and proliferation, reducing immune cell availability to this vital molecule (27, 28). Another crucial element in controlling immune cell function is the expression levels of metabolites such as fatty acids, branched-chain amino acids, and their transport proteins on the cell surface (29). The substantial reliance of AML cells on the NAD+ recycling pathway, which activates the DNA damage repair network to withstand immunotherapeutic stress, is particularly noteworthy. Furthermore, Myeloid-derived suppressor cells (MDSCs) are important immunosuppressive cells in the AML microenvironment (30, 31). By improving glycolysis and fatty acid metabolism, MDSCs sustain their life and function.

**Figure 1:**
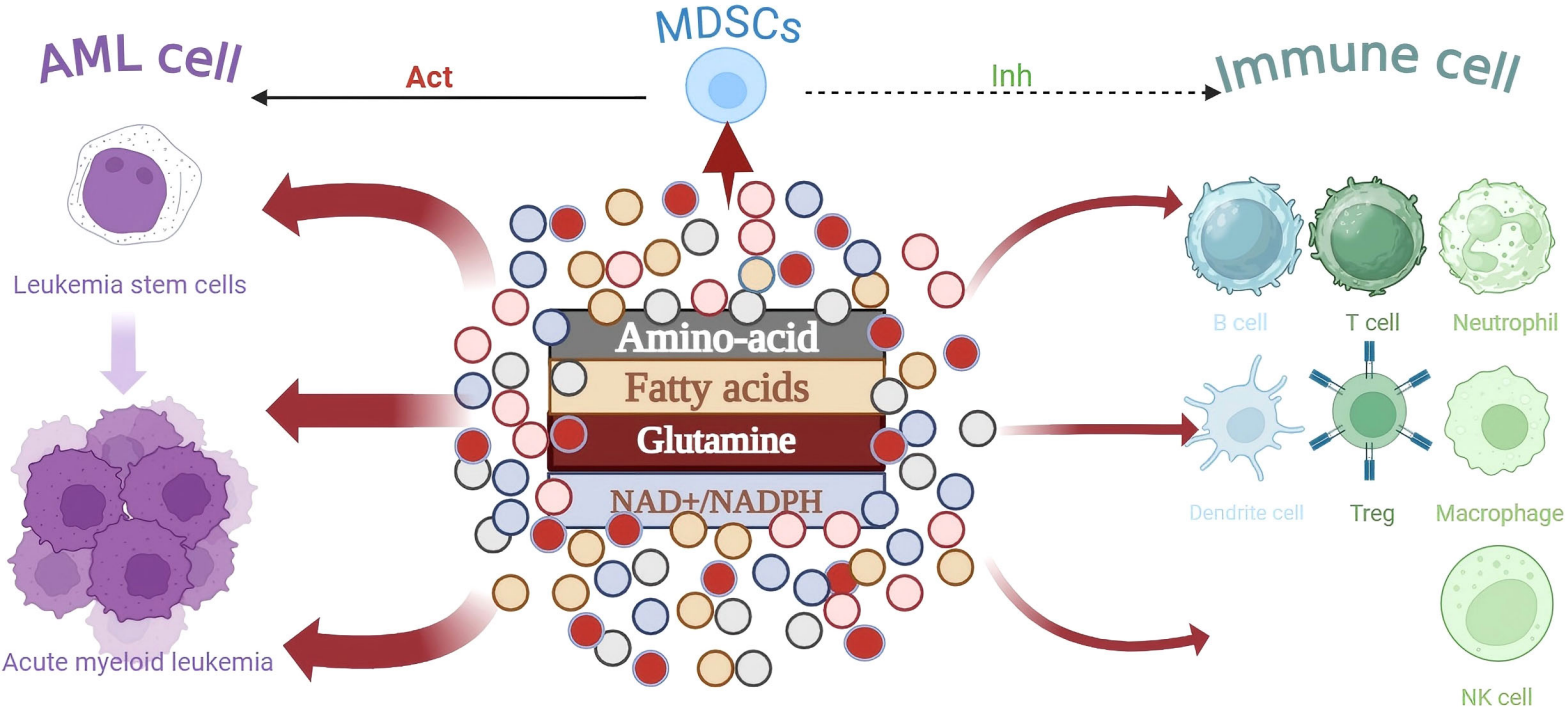
During the transformation of LSC cells into AML cells, the metabolism of immune cells is influenced by the competitive depletion of key nutrients, leading to immune escape.As the core immunosuppressive population, MDSCs inhibit the effector function of immune cells through metabolic pathways and work in synergy with AML cells to shape the immunosuppressive microenvironment.

The expression levels of metabolites such fatty acids, branched-chain amino acids, and their transport proteins on the cell surface are also important factors in regulating immune cell function. It is also notable that AML cells significantly depend on the NAD+ recycling pathway, which triggers the DNA damage repair network to endure immunotherapeutic stress. In the AML microenvironment, MDSCs are also significant immunosuppressive cells. MDSCs maintain their longevity and function by enhancing fatty acid metabolism and glycolysis (32). AML cells are more inclined to compete for glucose and amino acids, while MDSCs focus on fatty acid metabolism. Glycolysis in AML cells produces lactic acid, which is taken up by MDSCs as a FAO substrate. The cytokines secreted by MDSCs (such as IL-6) promote the survival of AML cells. This differentiation reduces direct nutritional conflicts (33) (**Figure 1**).

## 3 Impact of metabolic reprogramming in acute myeloid leukemia on the immune system

In AML, metabolic reprogramming has a multifaceted effect. Through competitive nutrient acquisition, AML cells not only accomplish metabolic remodeling, but their released metabolites also dramatically impair immune system function. This bidirectional regulatory system promotes favorable circumstances for AML’s immunological escape and speeds up the remodeling of the TME of AML. Notably, AML metabolic reprogramming affects immune cell function systemically at three levels: metabolites, signaling pathways, and essential enzymes.

### 3.1 Impact of metabolites on the immune microenvironment in acute myeloid leukemia

#### 3.1.1 Lactate

Even in oxygen-rich environments, tumor cells absorb glucose and use the glycolytic pathway to create lactate (34). The highly expressed MCT1 transporter protein releases this lactate into the TME of AML once it first builds up in AML cells. An acidic TME of AML is ultimately produced by the combination of lactate and hypoxic circumstances (35). Interestingly, in AML cells, lactate delivered by MCT1 also acts as a backup energy source for mitochondrial respiration. AML cells’ ability to invade and spread is further aggravated by this acidic (low pH) environment (36, 37).

MCT belongs to the SLC16A gene family (a member of CTs) and is a proton-coupled cotransporter responsible for transmembrane transport of monocarboxylic acids such as lactic acid and pyruvate. In AML cells, MCT1 (SLC16A1) can mediate lactic acid transport and also take up lactic acid to support OXPHOS and maintain energy (38). MCT4 (SLC16A3) is responsible for the efficient excretion of lactic acid. Its high expression in AML cells is the key to maintaining a high glycolytic rate and avoiding intracellular acidosis (39). AML upregulates both, pumps out lactic acid and protons, reduces the pH of bone marrow interstitial fluid, and creates an acidic microenvironment (40).

As demonstrated by lower glucose uptake, suppression of glycolytic processes, and decreased release of T cell components like IFN-γ, lactate buildup suppresses T cell function and encourages tumor immune escape (41). Through the lactate transporter protein, MCT1, Tregs cells can metabolize lactate as an alternative carbon source in the glucose-deficient environment caused by the malignant proliferation of AML cells, sustaining their proliferation and function (42). By triggering the NFAT15 signaling pathway in Tregs, this mechanism not only increases the immunosuppressive impact but also upregulates PD-1 expression. Interestingly, CD4+ T cell senescence and CD4+ and CD8+ T cell depletion were positively connected with the increase of PD-1+ Treg cells (43).

Chronic lactate exposure in the acidic bone marrow milieu inhibits the effects of CD16-mediated ADCC while promoting the differentiation of NK cells to CD56bright-regulated subpopulations via the GPR81 signaling pathway. Lactate buildup also inhibits the maturation of bone marrow DCs and the release of cytokines, which results in dysfunctional antigen presentation. Additionally, excessive lactate consumption causes NK cells to become intracellularly acidified, which hinders IFN-γ release and further reduces its ability to kill. Likewise, lactate buildup causes macrophages to produce more arginase 1, which helps them transition to an inactivated state (44).

Acidification brought on by lactate buildup in the TME severely impairs immune cell function, interferes with immune monitoring systems, and eventually encourages tumor immune escape. AML patients may benefit greatly from targeted therapies that target lactate metabolism in order to end the tumor-immune microenvironment loop. To reverse the acidic microenvironment and restore anti-tumor immune function, specific techniques include the creation of MCT1 inhibitors (e.g., AZD3965) to block lactate efflux or the manipulation of the activity of key enzymes of glycolysis (e.g., LDH) to inhibit lactate generation. Although restoring a normal pH environment may be beneficial to T cell function, further research is needed to determine whether MCT inhibitors have a direct impact on the metabolism and function of T cells themselves (45). Notably, PD-1/PD-L1 inhibitors, chimeric antigen receptor T cell (CAR-T cell) treatment, or DC vaccines may work in concert with lactate metabolism management to overcome the immunosuppressive milieu and greatly improve therapeutic efficacy (42). Future research should concentrate on examining the processes by which lactate metabolism and mitochondrial respiration are cross-regulated, with a particular emphasis on the functions of important nodes such as the succinate dehydrogenase complex.

#### 3.1.2 ROS

In order to preserve intracellular redox equilibrium, which is strictly controlled and necessary for the development and multiplication of healthy cells, reactive oxygen species (ROS) are important byproducts of mitochondrial oxidative metabolism. Reprogramming of metabolic and signaling pathways in AML cells results in excessively high ROS levels, which set off oxidative stress and genomic instability (46). It has been demonstrated that the mitochondrial respiratory chain and NADPH oxidase (NOX) are the primary sources of endogenous ROS in AML cells, whereas the accumulation of ROS in LSCs is further aggravated by the dysregulation of antioxidant response factors (GLRX, GPX, PRDX, and GSR). Notably, LSCs survival and colonization are aided by modestly higher ROS levels, despite the fact that they are harmful to the majority of cells. This dual-edged action may cause oxidative damage and ROS-dependent cell death in addition to activating oncogenic signaling pathways to advance AML. The fact that autophagy, a key target for controlling ROS, can prevent ROS overproduction and reduce the danger of DNA damage and carcinogenic mutations is especially concerning. However, excessive autophagy can also result in cell death (47).

ROS impact the immune system show intricate, two-way regulation characteristics (48) activating downstream molecules like NF-κB and MAPK in the T-cell receptor (TCR) signaling pathway and inducing the release of important cytokines like IFN-γ and TNF-α, physiological amounts of ROS can stimulate T-cell activation and proliferation. In the meantime, ROS increased the ability of cytotoxic T lymphocytes (CTLs) and natural killer (NK) cells to kill, stimulated T-cell activation, and improved the function of antigen-presenting cells (APCs). Interestingly, ROS is produced by activated T cells, M1-type macrophages, neutrophils, and NK cells, which leads to a more efficient removal of AML cells (49).

However, too much ROS buildup causes lactic acid buildup, the acidity of the microenvironment, and hypoxia, which causes resting T-cells and M2-type macrophages to undergo apoptosis. This suppresses the immune system’s reaction to AML and encourages immunological escape (50). It has been discovered that this disease frequently coexists with a compensatory increase in antioxidant capacity, indicating that immunological imbalance and elevated ROS levels may work together to cause AML (51).

The pathophysiology and management of AML are both impacted by ROS. In addition to efficiently eliminating AML cells, the body’s anti-tumor immune response can be triggered by carefully controlling ROS levels or specifically preventing ROS production. Nonetheless, developing trustworthy ROS-related biomarkers and precisely controlling ROS levels continue to be major obstacles in AML treatment. To improve the clinical outcomes for AML patients, future research should concentrate on examining the molecular mechanisms behind the ROS signaling pathway and creating safer and more focused ROS regulation regimens.

#### 3.1.3 Glutamine metabolism

Prior to the activation of glycolysis, glutamine, a crucial energy source for the growth and proliferation of AML cells, stimulates the cellular oxidative phosphorylation process through its metabolites. Interestingly, glutamine also controls the death of AML cells. It is converted to leucine by the SCLZA5/SCL3A2 bidirectional transporter, which then activates Ras-related GTPase to control the amino acid/Rag/mTORC1 signaling pathway. Apoptosis is finally triggered by this mechanism, which results in glutamine suppression of mTORC1 signaling targets (52). Glutamine is essential for the differentiation and operation of immune cells. Through the glutamine metabolic pathway, T cells produce ornithine and polyamines, such as putrescine and spermidine, which are necessary for sustaining intracellular DNA and protein synthesis. Activated T cells were found to increase glutamine absorption when compared to the resting condition (53).

However, glutamine is frequently competitively and malignantly absorbed by tumor cells in the TME, which prevents T cell differentiation. In particular, the differentiation of primary T cells into Tregs and Th17 cells is regulated by the glutamine-UDP-glcacc-N-glycan branching route in conjunction with the integrated stress response (ISR). Notably, *in vitro* studies demonstrated that memory CD8+ T cell development is facilitated by limiting glutamine supply. On the other hand, glutamine deprivation caused mitochondrial malfunction and death while suppressing the production of granzyme B (GzmB) and perforin-1 (PRF1) in CD8+ T cells.

KIT mutations benefit MDSCs in immunosuppressive cells by improving glutamine metabolism, which uses arginase 1 (ARG1) to remove arginine from the microenvironment and prevent T cell proliferation. Furthermore, glutamine metabolism serves as both a crucial regulator and a source of metabolic support in the activation of macrophages (54). Increased glutamine use dramatically lowers the rate of immune cell death, according to studies.

Significantly, glutamine deprivation causes Treg cells to become more abundant and encourages the release of immunosuppressive cytokines including TGF-β and IL-10, which both reduce M1-type macrophage activity and encourage M2-type polarization. Therefore, either restoring the amino acid content in the TME of AML or targeting and blocking the glutamine metabolic pathway in AML cells can effectively restore the killing activity of immune cells (55).

An inventive approach to treating AML is to target the glutamine metabolic pathway. A thorough investigation into the mechanism behind Gln’s function in AML immunoregulation will greatly improve the therapeutic outcome (56). Therapy that targets glutamine metabolism, a crucial regulator of AML and immune metabolism, has a lot of potential. Future studies should concentrate on 1) clarifying how glutamine metabolism works in concert with fatty acid metabolism, glycolysis, and other pathways; and 2) creating extremely effective and targeted medications. These discoveries will accelerate the development of immunometabolic treatment for AML.

### 3.2 Impact of AML metabolic signaling pathways on immune regulation

#### 3.2.1 PD-1

A crucial immune checkpoint receptor, PD-1 (programmed cell death protein 1) is mostly expressed on the surface of T cells, Tregs, and B cells. By attaching itself to the ligands PD-L1/PD-L2 found on the surface of dendritic cells (DCs) and macrophages, PD-1 can successfully prevent the immune system from becoming overactive. However, through a metabolic reprogramming mechanism, tumor cells transform this protective signaling system into a strategy for sustaining an immunosuppressive TME, which in turn promotes tumor proliferation (57).

Lactate buildup and STAT5 and PD-L1 expression in the bone marrow of individuals with newly diagnosed AML have been reported to be significantly positively correlated in clinical studies48. Notably, individuals with IFN-γ-stimulated complete remission (CR) and those with relapsed/refractory (R/R) AML had considerably higher levels of PD-L1 expression. By triggering the Akt/mTOR pathway, PD-L1-mediated reverse signaling can improve the glycolytic metabolism of PD-L1-positive tumor cells, hence speeding up tumor growth, according to mechanistic research. According to recent data, AML primitive cells can directly stimulate leukemogenesis by using PD-L1 to activate the CD274/JNK/Cyclin D2 signaling pathway. On the other hand, PD-L1 loss causes G1 phase cell cycle arrest and a marked decrease in LSCs.

Notably, when co-cultured with recombinant human PD-L1, PD-1-positive CD71+ red lineage progenitor cells and PD-1-positive CD34+ hematopoietic stem progenitor cells (HSPCs) activate caspase-3 in myelodysplastic syndromes (MDS)-pre-AML lesions, resulting in apoptosis and ineffective hematopoiesis (58).

The main manifestations of PD-1-mediated immunosuppressive effects were high Treg activation, upregulation of CTLA-4 expression, and enhancement of immunosuppressive functions. When paired with PD-L1, PD-1 blocked the conduction of molecules downstream of the TCR signaling pathway by phosphorylating intracellular tyrosine residues, which inhibited cytokine secretion and effector T-cell proliferation and function (59).49 Notably, PD-1 not only induces leukemia-specific CTL-mediated cytolysis but also interferes with IFN-γ-mediated cytotoxic effects through STAT3- and caspase-7-dependent pathways (60).

Additionally, PD-1 synergistic effects with immune checkpoint receptors like T cell immunoglobulin and ITIM domain (TIGIT) significantly enhance Tregs function and promote T-cell apoptosis, which together leads to the formation of a T-cell depleted state. PD-1 inhibitors have clinical potential in the treatment of AML, but they are limited by metabolism-mediated immune escape and biomarker loss. Currently, there are no clinically verified AML-specific PD-1 response markers. In future research, efforts should be focused on understanding the PD-1-driven T-cell metabolic reprogramming mechanism and developing combined immune-metabolic strategies. And patients are precisely stratified through multi-omics techniques, such as immune-metabolic synergy (PD-1 inhibitors + glycolytic/FAO inhibitors, SPHK1 inhibitors), and multi-target checkpoint blockade (PD-1 combined with TIM-3/TIGIT/VISTA inhibitors). Breaking through these bottlenecks will promote the transformation of PD-1 targeted therapy from the laboratory to clinical practice, and ultimately improve the prognosis of AML patients.

#### 3.2.2 mTOR

One essential intracellular signaling mechanism that controls cell growth, proliferation, metabolism, and survival is the mammalian target of the rapamycin (mTOR) pathway. According to research, 50–80% of patients with AML exhibit aberrant mTOR pathway activation. This not only encourages tumor cells to take up glucose and produce glycolysis, which speeds up tumor growth, but it also has a major impact on immune cell activity, including tumor-associated macrophages (TAMs), by altering the expression of cytokines, chemokines, and membrane receptors like PD-1 (61, 62).

The mTOR pathway is involved in the development of leukemia in two ways: first, it prevents leukemia cells from becoming normal blood cells by preventing cell differentiation; second, it encourages protein synthesis and cell growth, which keeps leukemia cells alive and enables them to proliferate. Interestingly, in FLT3-ITD patients, LSCs were uniquely linked to the mTOR signaling pathway.

Regarding immune regulation, the mTOR pathway controls the expression of immunological checkpoint molecules in addition to influencing the metabolic status of immune cells. mTORC1 inhibitors have been demonstrated to cause immune cells to switch from glycolysis to fatty acid oxidation, leading to improved metabolic adaptations and an extension of T-cell survival (63). Attenuated activation helps to mitigate mTOR-mediated NK cell metabolic and functional abnormalities in the early stages of AML. However, NK cell metabolic and functional failure results from chronic cytokine stimulation, particularly IL-15/mTOR signaling, in mice models of leukemia.

Furthermore, T cell function and depletion are caused by the upregulation of immune checkpoint receptor expression in AML patients and animal models (e.g., CTLA-4, PD-1, Tim-3). AML cells may avoid immune surveillance by a phenomenon known as cell exocytosis, which was notably boosted in clusters of cells triggered by the mTOR signaling pathway (64). Additionally, it was discovered that the mTOR pathway was activated by hypoxia in the AML microenvironment, and that the degree of hypoxia was positively connected with the activity of the mTOR signaling pathway (65). Patients with high-risk AML who expressed more hypoxia-associated genes were more sensitive to mTOR inhibitors (66, 67).

In addition to directly controlling the energy metabolism of tumor cells, the mTOR signaling pathway also controls immune cell activity, which has synergistic anti-tumor effects. There are still significant research gaps in current studies in revealing the specific dynamic changes of mTOR pathway activity, downstream phosphorylation signals, and metabolic crosstalk with immune cells at each stage of AML disease progression (initial diagnosis, remission, and recurrence). In particular, the lack of longitudinal and multi-omics research data on patient-paired samples limits our in-depth understanding of the immune escape mechanism of AML and the development of stage-specific treatment strategies. Future research needs to focus on three aspects to fill the gaps: 1. Longitudinal multi-omics studies: Paired cells from AML patients are collected at multiple time points, and mTOR signaling and metabolic dynamic maps are drawn through high-throughput technology; 2. Construct a three-dimensional co-culture *in vitro* model (including bone marrow matrix, AML and immune cells); 3. Integrate multi-omics data and functional verification to identify metabolic targets related to mTOR in key disease stages.

#### 3.2.3 AMPK

AMP-activated protein kinase(AMPK), is a key modulator of energy metabolism in cells. By phosphorylating important target proteins, including mTOR complex 1 (mTORC1), this crucial enzyme is sensitive to changes in intracellular ATP levels. When energy levels are low, AMPK is quickly activated to precisely regulate glycolysis, lipid metabolism, and mitochondrial function, promoting catabolism and inhibiting anabolism while achieving a rebalancing of cellular energy homeostasis (68, 69).

The AMPK signaling pathway is intimately linked to the proliferation and survival of AML cells. According to studies, AMPK regulates the metabolic homeostasis of immature AML cells to have a particular inhibitory impact, and its absence greatly increases the resistance of AML cells to chemotherapeutic medications. By activating AMPK, GSK621 was shown to be able to cause immunogenic cell death in AML cells, hence enhancing anti-tumor immune responses. Furthermore, AMPK can enhance the mitochondrial sensitivity of AML cells to the BCL2 inhibitor vinatocet and trigger the stress response via the PERK-ATF4 pathway. According to clinical data, people with AML who have lower levels of AMPK expression have a much lower chance of surviving. In solid tumor investigations, AMPK blocked β-catenin trans-activation via activation-dependently inhibiting AKT-mediated phosphorylation of the β-catenin S552 site, which in turn reduced the transcript levels of CD274 (PD-L1) in cancer cells (70).

The AMPK α1 subunit is essential for controlling the process by which IL-10 primarily mediates macrophages’ transition to an anti-inflammatory M2-like state. By suppressing T cell anti-tumor activity through the release of immune-suppressing proteins like IL-10 and IL-4, the M2-like TME promotes tumor growth by relying on oxidative phosphorylation (OXPHOS) and fatty acid oxidation (FAO). Notably, depending on how tumor cells and macrophages interact under various pathophysiological circumstances, the regulatory consequences of AMPK activation on macrophage polarization may vary considerably. These results unequivocally demonstrate the AMPK signaling pathway’s pivotal regulatory function in balancing energy metabolism and the immunological response to AML (71, 72).

Future research ought to concentrate on addressing the functional distinctions between immune and tumor cells’ AMPK subunits, particularly on clarifying the molecular processes by which the α1 subunit controls M2 polarization. In the meanwhile, more research is required to determine how the dynamic interchange of metabolites (such as lactate and lipid) in the TME affects the control of AMPK activation. These investigations will offer a crucial theoretical foundation for the creation of tailored medications that are particular to subunits and the execution of accurate microenvironmental interventions. With the deepening understanding of the AML immunometabolic network, AMPK-targeted therapy is expected to become a key strategy for precisely solving the problems of immunosuppression and drug resistance in AML, ultimately driving AML into a new era of combined metabolism-immune therapy.

### 3.3 Impact of metabolic enzymes on immunomodulation in acute myeloid leukemia

#### 3.3.1 IDH isocitrate dehydrogenase

About 20% of AML patients have functionally acquired mutations in isocitrate dehydrogenase (IDH), a crucial tricarboxylic acid cycle metabolic enzyme. The production of the oncogenic metabolite 2-hydroxyglutarate (2-HG), whose cumulative concentration can surpass the normal level by more than a thousandfold, is abnormally catalyzed by mutations in IDH1 (R132) or IDH2 (R140/R172), which results in altered enzyme activity and promotes leukemogenesis through a variety of mechanisms ^75. In^ particular, 2-HG, a structural analogue of α-KG, competitively inhibits TET2 and histone demethylases (e.g., KDM4C) to cause genome-wide DNA hypermethylation (CIMP) and abnormal histone modifications. This epigenetic condition causes differentiation-related genes like CEBPA and RUNX1 to be silenced while stem cell genes like HOXA/B and MEIS1 are persistently activated, ultimately leading to myeloid differentiation blockage (73).

Through a dual mechanism of action, 2-HG facilitates metabolic reprogramming that promotes the development of leukemia. On the one hand, 2-HG stabilizes HIF-1α and activates glycolytic metabolism by inhibiting prolyl hydroxylase (PHD); on the other hand, it causes mitochondrial dysfunction by inhibiting succinate dehydrogenase (SDH) (74). By acidifying the environment and creating an immunological escape barrier, this metabolic transition not only increases LSCs’ potential for self-renewal but also prevents T-cell activation and NK cytotoxicity (75). Furthermore, via upregulating BCL-2 family proteins like MCL-1, mutant IDH confers apoptosis-resistant characteristics. Significantly, clinical research has demonstrated that IDH mutations frequently work in concert with co-mutations such as FLT3-ITD and NRAS, which collectively stimulate the RAS/MAPK and PI3K/AKT signaling pathways, hence facilitating the aberrant survival and proliferation of AML cells (76).

The advent of IDH inhibitors is a major milestone in the field of AML targeted therapy, verifying the targeting of the consistent disease axis from gene mutations to metabolic abnormalities and epigenetic regulation. However, the limitations of monotherapy are becoming increasingly prominent, and drug resistance is the core problem that needs to be urgently addressed. In the future, the synergistic effects of IDH inhibitors with new-generation epigenetic regulators (such as EZH2 and DOT1L inhibitors) should be systematically evaluated. And develop nano-delivery systems to penetrate stem cell niches, establish a dynamic 2-HG monitoring system, analyze the interaction between IDH mutations and chromatin remodeling complexes (such as SWI/SNF), thereby constructing a metabolomics-guided precision medication model.

#### 3.3.2 IDO (indoleamine 2, 3-dioxygenase)

One significant intracellular monomer enzyme involved in the development of many malignancies is IDO. It encourages the development, invasion, and metastasis of cancer cells in addition to inhibiting immune system activity. By suppressing anti-tumor immune responses and causing tumor treatment resistance, IDO has been shown in recent research to have a substantial impact on the course of cancer (77). A persistent inflammatory response in the TME of AML in AML triggers type 2 MSPC, which suppresses the immune system and secretes excess IDO (78).

IDO primarily plays a biological role in the conversion of the vital amino acid L-tryptophan to L-kynurenine. The two immunosuppressive effects of this metabolic process are as follows: tryptophan deficiency causes T-cell cycle arrest, while kynurenine and its metabolites directly poison T cells and natural killer cells (NKCs), preventing T-cell proliferation and encouraging the development of Tregs (79).

There are two known isoforms of IDO: IDO1 and IDO2. IDO2 is expressed selectively, mostly in particular cell subsets such as antigen-presenting cells. Research has indicated that by promoting IDO1-mediated Tregs function, IDO2 may play a role in adaptive immune control. By means of processes like stimulating Treg cell proliferation and encouraging the transformation of M0-type macrophages into M2-type, IDO creates an immunosuppressive milieu in the TME that facilitates tumor evasion of immune surveillance (80).

IDO1 plays a key role in immune escape in AML, but targeted therapy is still in the early stage of exploration. The core challenges include the lack of AML-specific clinical data, limited efficacy of monotherapy, and concerns about the safety of combination regimens. Future breakthroughs can rely on three aspects: precise identification of IDO1-dependent AML subpopulations (such as single-cell sequencing to analyze IDO1 + cell clusters), development of novel metabolic intervention strategies (such as regulating the availability of tryptophan through gut microbiota), and exploration of time-dependent dosing regimens (such as first targeting leukemia cells and then reversing immunosuppression). The value of IDO1 inhibition is not limited to direct anti-tumor effects; it can also improve the immune microenvironment and create conditions for other treatments such as vaccines or cell therapies.

#### 3.3.3 Tyrosine kinase

TK plays a central role as a key signaling molecule in cell proliferation, differentiation, metabolic regulation and immune response. Based on their structural and functional characteristics, TKs can be divided into two major groups: receptor-type tyrosine kinases (RTKs) trigger downstream signaling cascades by specifically binding to growth factors (e.g., SCF, FLT3 ligands); and non-receptor-type tyrosine kinases (NRTKs), such as SHP-1 (PTPN6) and PRL2, are mainly involved in the negative regulation and cross-talk of signaling pathways. In the pathogenesis of AML, mutation or aberrant activation of TKs (e.g. FLT3-ITD) promotes the acquisition of drug resistance and immune escape by LSCs through the remodeling of the metabolic network and the immune microenvironment, ultimately resulting in the formation of a distinctive “metabolic-immune double barrier” (81).

Through metabolic reprogramming, aberrant TK activation in AML gives LSCs a survival advantage. The main mechanisms of this process are as follows: Through the FLT3-STAT5 signaling axis, the FLT3-ITD mutation directly increases phosphofructokinase platelet-type (PFKP), which in turn promotes glucose uptake, lactate generation, and glycolysis, all of which encourage the rapid proliferation of LSCs. In the meantime, glutaminase (GLS1) production is driven by the PI3K/AKT/mTORC1 pathway, which sustains mitochondrial oxidative phosphorylation (OXPHOS) via the tricarboxylic acid cycle (TCA) (82). In order to preserve metabolic flexibility, prolonged FLT3 inhibitor treatment triggers the SPHK1/S1P-β-catenin pathway, which in turn up-regulates GLS1 and eventually results in drug resistance. Acetyl coenzyme A carboxylase (ACC1) is upregulated by KIT-PI3K/AKT signaling to facilitate fatty acid synthesis from scratch and improve membrane lipid fluidity and signaling efficiency in LSCs. TKs’ inhibition of the AMPK pathway lowers mitochondrial autophagy, which results in the buildup of damaged mitochondria and a decreased ability to withstand ROS. It also keeps LSCs in a low metabolic resting state (83).

**Figure 2:**
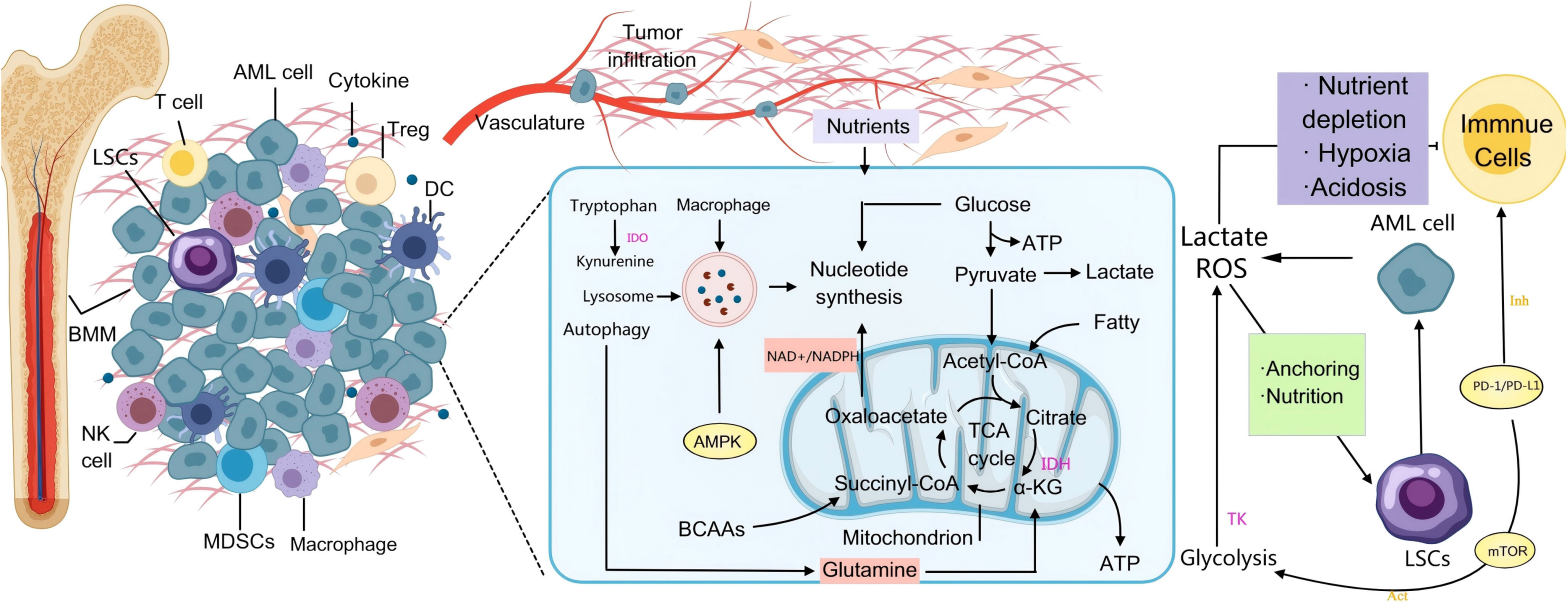
AML-mediated metabolic immune regulation in the BMM. On the left side, AML cells infiltrate the bone marrow and secrete cytokines that recruit immunosuppressive cells such as regulatory T cells (Tregs) and MDSCs, which collectively inhibit the anti-tumor activities of T cells and NK cells. This process establishes an “immune-tolerant microenvironment” that shields AML cells from immune-mediated elimination. In the central metabolic pathway, AML cells utilize the Warburg effect—enhanced glycolysis leading to lactic acid production—to rapidly generate ATP. Accumulated lactic acid induces microenvironmental acidosis, further dampening immune responses. Additionally, glutamine catabolism replenishes the tricarboxylic acid (TCA) cycle, supplying precursors for nucleotide and lipid biosynthesis. Mutations in IDH result in the oncometabolite 2-hydroxyglutarate (2-HG), which disrupts normal DNA methylation patterns and contributes to malignant transformation. Under conditions of nutrient deprivation, AMPK-mediated autophagy is activated to degrade cellular components and sustain leukemic cell survival. On the right side, PD-L1/PD-L1 on the surface of AML cells enhances their own glycolysis by activating pathways such as PI3K/Akt/mTOR (Warburg effect), while binding to PD-1 on the surface of T cells, inhibiting their glycolysis and amino acid metabolism, promoting their transformation to fatty acid oxidation, and ultimately leading to T cell failure. They also attach to stromal cells through adhesion molecules, thereby evading chemotherapy and immune surveillance. Activation of the mTOR signaling pathway enhances metabolic activity and stemness, contributing to therapeutic resistance and disease relapse in AML.

Interestingly, this metabolic resting state is reversed by SHP-1/PTPN6 suppression. By preventing AKT ubiquitination degradation, triggering β-catenin signaling, and promoting PFKP expression, SHP-1 deficiency improves chemosensitivity in the AKT-β-catenin-PFKP axis. This causes LSCs to transition from a low metabolic state to a high energy metabolism. By altering immunological checkpoint molecules and immune cell activity, TK facilitate immune escape. For instance, FLT3-STAT3 signaling directly binds to the PD-L1 promoter and stimulates its transcription, while PRL2 indirectly upregulates PD-L1 through the TLR4-NF-κB axis and suppresses the cytotoxic activity of T cells (84). TKs control immunological escape mechanisms and metabolic reprogramming in AML, creating a complicated web of vicious cycles. In addition to successfully reversing drug resistance in LSCs, targeted inhibition of these kinases and their downstream metabolic-immune critical nodes also changes the tumor immunological milieu, creating novel therapy options for AML treatment has shifted from single TKI inhibition to a dual-targeted combination of “metabolism-immunity”, which is expected to overcome drug resistance and improve therapeutic efficacy. In the future, efforts should be focused on analyzing the molecular interactions of the TK-metabolism-immune axis, establishing a dynamic biomarker monitoring system, and designing precise and individualized joint solutions. With the development of multi-omics technologies and novel inhibitors, TK-directed immunometabolic therapy may become a new pillar of precision medicine for AML (**Figure 2**).

## 4 Role of immune cell metabolic reprogramming in acute myeloid leukemia

The malignant progression and immune escape of AML are highly dependent on the spatiotemporal dynamic regulation of metabolic reprogramming, which is characterized by abnormal interactions among energy metabolism, nutrient competition and metabolite signaling networks. Studies have shown that certain metabolic pathways support certain immunophenotypes to a greater extent than others. Immune cell metabolism is regulated by AML cells hijacking metabolic pathways and the TME, forcing immune cells to use different pathways than normal to meet energy demand, further inducing that the phenotype of immune cells deviates from normal. Thus, a “metabolic-immune escape” network is formed. Although AML constructs an immunosuppressive microenvironment through metabolic reprogramming, immune cells can also launch a counterattack through metabolic adaptive adjustment. Novel approaches to AML treatment have been made possible by recent developments in metabolic processes and immunological checkpoints. The two main processes of metabolite regulation and metabolic pathway reprogramming will be the subject of this chapter.

### 4.1 Metabolic pathway reprogramming in immunotherapy for acute myeloid leukemia

#### 4.1.1 Glycolysis

Immune cells including neutrophils, M1 macrophages, dendritic cells, naïve T cells, and effector T cells use glycolysis as their primary metabolic pathway. In the activated state, neutrophils mostly rely on aerobic glycolysis for energy because of their incredibly low mitochondrial concentration. Similar to this, when activated, the majority of lymphocytes—including inflammatory M1 macrophages—move from oxidative phosphorylation to the glycolytic pathway in order to fulfill their energy and biosynthetic demands (85). On the other hand, M2 macrophages and regulatory T cells have a tendency to reduce glycolytic activity and use the tricarboxylic acid cycle (TCA) in the activated state (86).

Two distinct functional subtypes of macrophages can be activated: the anti-inflammatory M2 type aids in wound healing, while the pro-inflammatory M1 type is high in lysosomal particles. Increased glycolytic pathways allow macrophages to survive in hypoxic conditions. When the M1 type is polarized by IFN-γ, aerobic glycolysis is significantly activated and oxidative phosphorylation is inhibited. This metabolic reprogramming makes M1 macrophages rely heavily on glycolysis for their survival. Notably, GPR132 and GPR65 receptors allow tumor-associated macrophages to detect lactate signals in the TME. These cells then encourage M2-type polarization and release pro-tumor growth factors such as L-proline and polyamines.

Within minutes of activation, dendritic cells (DCs) dramatically increase lactate generation and glycolytic activity, according to recent studies (87). Notably, human-derived dendritic cells with autotolerance characteristics generated a lot of lactate and showed an exceptionally active glycolytic metabolism. On the contrary, exogenous lactate prevents monocyte-derived dendritic cells (moDC) from differentiating and maturing. However, by controlling the generation of IFN-α, bone marrow-derived plasmacytoid dendritic cells (pDC) have anti-tumor properties. Recent research has verified that tumor-derived lactate does, in fact, have an immunomodulatory effect on pDC (88).

CD8+ and CD4+ T cells (including Th1, Th17, and Treg subpopulations) are the two primary subpopulations of T cells. The high-mobility family box protein (Tox) is a crucial nuclear component that controls CD8+ T cell depletion and malfunction, according to studies. Interestingly, inhibiting glycolysis increases the immunoreactivity of memory CD8+ T lymphocytes and encourages their production, while Tox expression is strongly correlated with inhibitory receptors like PD-1. Increased lactate levels in the TME of AML caused CD8+ T cells to produce the Tox protein (89). This mechanism impairs effector T cell function in addition to decreasing CD8+ T cell infiltration potential, indicating that Tox may be a crucial biomarker for assessing tumor treatment response.

From a metabolic standpoint, glycolytic enzymes activate the p70S6 kinase and eIF4E binding protein 1 signaling pathways, which are necessary for IFN-γ production in effector CD8+ T cells. Lactate, a raw ingredient for biosynthesis, is produced by activated cytotoxic T cells mainly through the glycolytic pathway. On the other hand, too much tumor-derived lactate prevents CD8+ T cells from expressing NFAT, which hinders the production of IFN-γ and eventually results in tumor immunological tolerance. These results imply that altering the glycolytic pathway can improve both the immune response against tumors and the tumor cells’ susceptibility to other therapies.

Furthermore, lactate can be transformed into pyruvate by LDHB to enter the TCA cycle after entering CD4+ T cells via the MCT1 transporter, hence negative feedback controlling the glycolytic activity of T cells (90).

Fatty acid oxidation is the main source of energy for memory T cells, in contrast to effector T cells. Conversely, T cells in the resting state use aerobic oxidative metabolism to fully catabolize glucose to carbon dioxide. The glycolytic metabolic pathway of T cells is markedly changed following antigenic stimulation: expression of LDHA and glucose transporter protein 1 (GLUT1) is elevated, glucose absorption is enhanced, and lactate generation is raised. To obtain the energy and biosynthetic raw materials needed for their quick proliferation, activated T cells resorted to the glycolytic or aerobic glycolytic pathway to convert glucose to lactate.

Tregs have limited glycolytic usage and rely mostly on fatty acid oxidation for energy. Nevertheless, it was discovered that Tregs tended to use the glycolytic route during activation. The variety of Treg cell subpopulations and the tissue microenvironment may be the cause of this variation in metabolic preference. Tregs regulate the immune system in the tumor immunological milieu by preventing effector T cells from cytotoxically attacking the tumor. Consequently, the immunotherapeutic effectiveness of different malignancies is significantly influenced by the ratio of Tregs to effector T cells.

The proliferation of NK cells and their antiviral and anticancer properties are significantly influenced by LDHA-mediated aerobic glycolysis. Research has demonstrated that oxidative phosphorylation and glycolysis cooperate to preserve the functional homeostasis of NK cells. Notably, by compromising mitochondrial activity, lactate in the TME can strongly encourage early trNK cell apoptosis (91).

By modifying immune cell functional polarization and lactate-mediated microenvironmental immunosuppression, glycolysis is a crucial center of immunological-metabolic control in AML, which has a substantial impact on the course of the illness and the effectiveness of treatment. In order to create precise treatment plans to address metabolic heterogeneity, future research should concentrate on integrating immunotherapy strategies with metabolic regulation. Additionally, it should thoroughly examine the mechanism of the lactate-mediated metabolism-immunity interaction in order to create new opportunities for AML immunotherapy.

#### 4.1.2 OXPHOS

Functional mitochondria are essential for the survival of cells in AML. Increased mitochondrial mass, increased mitochondrial DNA (mtDNA) copy number, aberrant reliance on oxidative phosphorylation (OXPHOS), increased mitochondrial biosynthesis, and changes in the translation system without corresponding increases in respiratory chain complex activity are some of the distinctive mitochondrial features of AML cells in comparison to normal hematopoietic progenitor cells (92). As a crucial link between catabolism and anabolism, mitochondria not only produce ATP to meet the energy needs of the cell, but they also incorporate glucose metabolites to supply a carbon source for the synthesis of biomolecules. need, but also incorporate glucose metabolites to supply a carbon source for the synthesis of biomolecules, turning into a crucial transitional phase between anabolism and catabolism. According to clinical research, vinblastine and azacitidine treatment dramatically lowers OXPHOS levels in LSCs, inhibits electron transport chain complex II activity, and disturbs the TCA cycle in AML patients (93).

Recent studies have revealed a key cross-regulatory mechanism: when mitochondrial function is impaired in AML cells, lactate metabolism is compensated to maintain cell survival. Multiple studies, especially one targeting BET inhibitor resistance, have found that AML cells exhibit a “lactic-driven vulnerability to respiratory metabolism” when electron transport chain Complex II (ETC Complex II, succinate dehydrogenase SDH) activity is inhibited. In this case, succinate from the TCA cycle accumulates upstream, while electron flow to complex III is blocked, leading to a dramatic decrease in mitochondrial respiration and ATP production. At this point, AML cells take up a large amount of exogenous lactate by upregulating the expression of MCT1 (35).

Research has demonstrated a significant correlation between the chemoresistance of AML cell lines to cytarabine (AraC) and their levels of oxidative phosphorylation (OXPHOS). Targeting suppression of the electron transport chain, the fatty acid oxidation route or mitochondrial protein synthesis greatly increased AraC’s anti-leukemic efficacy (94). Furthermore, by combined suppression of the OXPHOS and/or purine ab initio pathway, the isoflavone ME-344 significantly increased the effectiveness of vinatocetin in the treatment of AML in both *in vitro* and *in vivo* models (95). Targeting the electron transport chain, fatty acid oxidation pathways, or mitochondrial protein synthesis also significantly increased vinatocetin’s efficacy in treating AML.

The main energy sources for immune cells such as memory T cells, M2 macrophages, and Tregs are mitochondrial-centered OXPHOS and FAO (23). Metabolically quiescent naïve CD4+ T cells are the only T cell subpopulation that depends on OXPHOS for energy (83). The metabolic pattern of T cells changes from OXPHOS to glycolysis during activation. Through the extracellular signal-regulated kinase (ERK) pathway, CD28 co-stimulatory signaling stimulates glycolysis, which speeds up this metabolic transition and controls T cell proliferation and differentiation.

In order to stimulate forkhead box protein P3 (FOXP3) transcription factor expression, cellular differentiation, and the production of the anti-inflammatory cytokine IL-10, Treg cells need high amounts of ROS and OXPHOS. As long-lived effector cells, Th17 cells rely on mitochondrial OXPHOS to function *in vivo*. By preventing the breakdown of apoptosis regulators caused by mitochondrial autophagy, OXPHOS increases the apoptosis resistance and survival of Th17 cells. Th17 cell viability and apoptosis resistance. OXPHOS is also necessary for the polarization of CD8+ Tc22 cells (96). Through the HIF-1α and aromatic hydrocarbon receptor (AhR) pathways, coenzyme A (CoA) triggers Tc22 cell differentiation and OXPHOS activation when polarizing cytokine stimulation is not present. Notably, the body’s reactivity to PD-L1 blocking therapy was markedly improved by the CoA precursor pantothenate.

Clinical observations revealed that CD8+ T cells with high OXPHOS activity tended to be resistant to immunotherapy. This could be because overactivity causes T cells to become depleted, which reduces their capacity to kill tumors (97). On the other hand, low OXPHOS levels result in impaired immune cell capabilities, including a reduction in T cell proliferation and killing ability. Consequently, the exact control of OXPHOS levels is essential for immune cell reactivation (98, 99).

The intact TCA cycle serves as a crucial substrate for the electron transport chain (ETC) complex, and the M2 subpopulation in macrophages primarily depends on oxidative phosphorylation (OXPHOS) for energy. 87 The expression level of the serine/threonine kinase GSK3β can be controlled by IL-15-expanded NK cells or NK cells obtained from AML patients (100). This greatly improves NK cell maturation and anti-tumor effectiveness. Additionally, by selectively blocking the OXPHOS pathway in tumor cells, NK-128 successfully increased AMP-activated protein kinase (AMPK) signaling.

The cross-regulation between lactic acid metabolism and mitochondrial respiration constitutes the core network for AML cells to adapt to the microenvironment, maintain proliferation and evade treatment. Lactic acid plays a triple role in this network: (1) It serves as a crucial metabolic fuel, providing power for mitochondria; (2)Important signaling molecules, shaping the immunosuppressive microenvironment; (3) Novel epigenetic substrates that directly regulate gene expression through histone emulsification.

#### 4.1.3 Fatty acid oxidation

Through metabolic reprogramming, AML cells have a strong reliance on fatty acid oxidation (FAO) and fatty acid synthesis, a metabolic signature that is essential for their quick growth, energy provision, and biosynthesis. FAO is the primary mechanism for ATP synthesis in AML cells, as opposed to glucose metabolism. Important fatty acid metabolizing enzymes such as CPT1A and ACSL showed markedly elevated expression levels, which makes them extremely potential therapeutic targets (101).

Four primary pathways contribute to the improvement of metabolic efficiency in the fatty acid oxidation (FAO) reprogramming mechanism of AML cells: upregulation of key enzyme expression, enhancement of fatty acid uptake, preservation of enzyme stability, and utilization of microenvironmental resources. Tumor-associated macrophages (TAM) are specifically affected by CD36-mediated lipid over-uptake, which results in oxidative phosphorylation failure, lipid droplet accumulation, and M2 phenotypic polarization (102). Regarding the regulation of important enzymes, ACSL (long-chain fatty acid acyl-coenzyme A ligase) is in charge of activating fatty acids involved in oxidative metabolism, while CPT1A (carnitine palmitoyltransferase 1A) facilitates fatty acid entry into the mitochondrion for β-oxidation.Transporter proteins like CD36 are increased in the fatty acid absorption pathway, which improves the active uptake of exogenous fatty acids. Furthermore, it is possible to sustain the breakdown of enzymes like CPT1A by preventing their stability and extending metabolic activity. AML cells can receive exogenous fatty acids from bone marrow adipocytes and MSCs in order to meet FAO needs in terms of microenvironmental resources.

Regarding the reprogramming of fatty acid synthesis, AML cells trigger the fatty acid synthesis pathway by upregulating important synthases like ACC (acetyl coenzyme A carboxylase) and FASN (fatty acid synthase), which catalyze the production of precursors like malonyl coenzyme A, creating a “metabolic double-edged sword” effect (103). It is important to note that AML cells exhibit exceptional substrate integration skills during metabolic reprogramming. They can store excess fatty acids as lipid droplets to dynamically balance the needs of synthesis and catabolism, and they can use acetyl coenzyme A, which is produced by glycolysis, as a synthetic raw material. During metabolic reprogramming, AML cells exhibit exceptional substrate integration capabilities. They can store excess fatty acids as lipid droplets, dynamically balancing the demands of catabolism and synthesis, and they can use acetyl coenzyme A, which is produced by glycolysis, as a raw material for synthesis, achieving synergistic glycolipid metabolism. Multiple supports for the malignant development of AML are provided by this reprogrammed fatty acid metabolic network: supplying the necessary basic ingredients for the production of membranes;encouraging the synthesis of lipoproteins to sustain biomass accumulation and signaling, and accelerating cell proliferation through the FAO’s effective ATP generation. The synergistic impact of FAO with synthetic pathways gives AML cells a great deal of metabolic flexibility, especially in nutrient-poor microenvironments (104). The regulation of immune cell activity, differentiation, and adaptation is significantly influenced by fatty acid production and fatty acid oxidation (FAO). FAO plays an important immunomodulatory role even though it is not a prominent metabolic pathway in the majority of immune cells.

FAO serves as a vital metabolic foundation for memory T cells’ long-term survival and quick antigen response by supplying energy and preserving mitochondrial activity, particularly for CD8+ Tm. Tm synthesis and function were not substantially impacted by the deletion of CPT1A, a crucial FAO enzyme, indicating the possibility of additional metabolic compensation mechanisms. Since effector T cells (Teff) is mostly dependent on glycolysis, FAO suppresses Teff activation, increases PD-1 expression, and decreases IFN-γ production (105). High amounts of FAO stimulate the development of Tregs, which are less glycolytically active and improve immunosuppressive function; however, genetic research indicates that FAO may not be the main energy source for Treg. Furthermore, different types of fatty acids have distinct impacts on T cell function. For example, short-chain fatty acids (SCFA) suppress inflammatory responses and enhance Treg differentiation and IL-10 secretion, while long-chain fatty acids (LCFA) activate Teff proliferation but inhibit Treg function (106).

There is geographical variation in the metabolic regulation of B cells. While light zone B cells (LZ) prefer glycolysis to adjust to antigen presentation and affinity screening requirements, dark zone B cells (DZ) in the germinal center (GC) rely on FAO to support proliferation and differentiation, which may be related to the nutrient-competitive microenvironment.

While M2-type macrophages effectively produce ATP via FAO in nutrient-deficient environments to promote immunological tolerance function, MZ-like macrophages rely on FAO to sustain mitochondrial function and anticancer activity. Notably, there is still a lack of comprehensive research on how FAO inhibition affects macrophage antitumor activity (107).

Because dendritic cells (DCs) are vulnerable to lipids in the TME, dysfunction results from excessive lipid levels (such as oxidized lipids), which hinder DCs’ capacity to present antigens cross-presentatively. Through the hijacking of FAO and fatty acid synthesis (FAS) metabolic pathways, tumor cells prevent DCs from migrating and secreting pro-inflammatory cytokines. In order to meet energy demands and preserve cytotoxic effects, NK cell activation causes a shift in metabolic patterns from glycolysis to FAO. Furthermore, in order to facilitate immunological escape, tumor cells alter the metabolism of immune cells (such as DC and Treg) by releasing fatty acids or oxidized lipids (19, 101). For tolerant cells (such as Treg and M2 macrophages) to continue to function in the absence of nutrients, they rely heavily on FAO’s effective ATP production.

Targeted research on the metabolic regulation mechanisms of distinct immune cell subpopulations is necessary, as studies have revealed notable variations in their reliance on fatty acid oxidation (FAO). Notably, FAO restriction may impair immunological-tolerant cells like M2-type macrophages and Tregs, which strengthens the body’s immune response against tumors (108). Blocking the intake of long-chain fatty acids or altering the kinds of fatty acids (e.g., by supplementing with short-chain fatty acids) can significantly change immune homeostasis. It is also anticipated that the tumor immunosuppressive milieu would be reversed by targeted intervention of important molecules involved in tumor lipid metabolism, such as CD36 and CPT1A.

### 4.2 Impact of metabolic reprogramming on immune regulation in acute myeloid leukemia

#### 4.2.1 Glucose

The ability of various cells to absorb glucose, a crucial substrate for glycolysis and mitochondrial respiration, varies greatly; myeloid cells have the highest capability, followed by T cells and cancer cells. Impairment of metabolic adaptations in T cells and suppression of metabolic functions in NK cells are the primary characteristics of glucose metabolism vulnerability in immune cells (109).

Glucose deprivation drives T cells to use oxidative phosphorylation-dependent (OXPHOS) in the AML milieu, but an overabundance of ROS in the mitochondria eventually causes death, which results in dysregulated glucose metabolism (110). Furthermore, miR-23a-3p carried by AML exosomes reduces mitochondrial biosynthesis and targets PGC-1α to decrease mitochondrial function, hence reducing the cell’s metabolic reserve capacity. Glycolysis is crucial for NK cell cytotoxicity, and in the AML microenvironment, glucose restriction dramatically lowers the synthesis of perforin/granzymes, which eventually results in the loss of NK cells’ ability to kill (111).

There are still several obstacles in the way of glucose-targeted treatments. Single-cell metabolomics is necessary to identify subclonal-specific vulnerabilities in the dynamically evolving metabolic profile of AML. Notably, systemic metabolic therapies may disrupt immunological homeostasis, underscoring the significance of creating delivery methods that target tumors, such as CD33 antibody-drug couplers. In order to develop novel epimetabolic combination therapies, future research should concentrate on clarifying the metabolic-epigenetic-immune regulatory network and exploring the impact of metabolites like α-KG and NAD+ on the epigenetic reprogramming of T cells.

#### 4.2.2 ATP

In immunotherapy for AML, ATP plays two regulatory roles. Anti-tumor action is greatly increased by higher ATP production, which serves as a functional fuel for immune cells. For instance, Venetoclax increases NK cells’ capacity to destroy AML cells by stimulating their mitochondrial metabolism, which in turn increases ATP generation. However, by lowering its level, ATP, the energy source of cancer cells, can directly cause apoptosis. Mitochondrial malic enzyme 2 (ME2) inhibitors have the ability to lower ATP levels in AML cells, which can cause an energy crisis and death. They can also lower NADPH production, which can increase reactive oxygen species and oxidative damage, and eventually have an anti-leukemia effect (112).

ATP, the primary source of cellular energy, is essential for immune cell activation and response. Research has demonstrated that a sufficient energy supply is necessary for T cell activation, effector function maintenance, and memory function. Notably, T cell activity can be compromised by AML cells through the inhibition of ATP synthesis and creatine uptake, which provides a crucial channel for their immunological escape (113).

By focusing on and suppressing SLC6A8 gene expression in T cells, miR-19a-3p delivered by sEVs was shown to dramatically lower creatine absorption and ATP levels. It is noteworthy that the NK cell subset (CD161lowCD218b+), which showed increased degranulation activity and cytokine release, was especially affected by Venetoclax’s metabolic modulatory action. Furthermore, the growth and immunosuppressive properties of AML cells were strongly linked to increased ATP levels (114).

In AML immunotherapy, the ATP-gated ion channel P2X7 receptor has a special dual regulatory role. By controlling Treg cell function, activation of this receptor suppresses the anti-tumor immune response in addition to encouraging AML cell proliferation and immune surveillance evasion. Interestingly, P2X7 receptor activation also makes AML cells more resistant to chemotherapy medicines; the likelihood of chemotherapeutic medications entering the cell is increased when non-selective membrane pores larger than 500 Da occur. According to recent research, the P2X7 receptor and its genetic variations hold promise as new AML diagnostics and treatment targets (115). Clinically useful new approaches to treating AML have been discovered based on the mechanism of ATP-P2X7 receptor interaction.

A vital and complex component of AML immunotherapy is ATP. Comprehensive research on the mechanisms governing ATP metabolism (such as P2X7 receptor activity) in conjunction with metabolic imaging tools like PET-CT that track the dynamics of ATP metabolism in AML and immune cells in real time can offer accurate direction for tailored treatment. In addition to optimizing the therapeutic efficacy, this focused regulatory approach dramatically raises the patient survival rate. Controlling the ATP level and altering the immunological milieu, is anticipated to further strengthen the anti-tumor immune response.

#### 4.2.3 Amino acids

The following are the main ways that abnormalities in amino acid metabolism encourage AML formation and immune escape: metabolism of branched-chain amino acids: Branched-chain amino acid transaminase 1 (BCAT1), which is particularly abundantly expressed in AML stem cells, catalyzes the breakdown of BCAAs to produce α-ketoglutarate (αKG), which promotes DNA hypermethylation and keeps leukemic stem cells (LSCs) self-renewing. Notably, in individuals with IDH mutant AML, BCAT1 overexpression was substantially linked to a poor outcome. Methionine metabolism: Through the SETD2-dependent histone H3K36 methylation pathway, methionine adenosyltransferase 2A (MAT2A) promotes the conversion of methionine to S-adenosylmethionine (SAM), which preserves leukemia-specific epigenetic characteristics (116). Tryptophan metabolism: Strong immunosuppressive metabolites that both directly reduce CD8+ T-cell activity and stimulate Tregs differentiation are produced by the IDO-activated kynurenine pathway (117).

Glutamate is a crucial carbon source for oxidative phosphorylation (OXPHOS), which is catabolized by glutaminase (GLS) to form α-ketoglutarate (αKG), which gives AML cells energetic support during the metabolism of non-essential amino acids. To counteract oxidative stress, cells absorb cysteine through the xCT transporter protein (SLC7A11) and use it to synthesize glutathione (GSH). Notably, iron death is brought on by cysteine deficiency and is intimately associated with the buildup of ROS (118). AML cells also need exogenous uptake to survive because of argininosuccinate synthase 1 (ASS1) deficiency.

The immunological milieu is modulated by amino acid metabolism, which has a substantial impact on the course of AML. Methionine restriction efficiently suppresses the proliferation of LSCs. Tryptophan metabolism via the IDO-mediated kynurenine pathway contributes to the immunosuppressive mechanism by promoting Treg differentiation via AhR signaling and suppressing T cell proliferation, so establishing an immunological escape milieu. In the meantime, AML cells’ production of arginase II causes the microenvironment’s arginine to be depleted, which inhibits T cell activity and encourages HSC proliferation (119).

By combining immunomodulatory and epigenetic intervention techniques, targeting amino acid metabolic networks greatly increases therapy efficacy and generates new concepts for treating AML. Future research ought to concentrate on the traits of metabolic heterogeneity and employ single-cell metabolomics to thoroughly examine each AML subtype’s metabolic susceptibility (e.g., the regulatory link between IDH mutations and BCAT1 expression). To successfully address the issue of drug resistance, combination treatment plans may take into account epigenetic medications (like HDAC inhibitors) or mitochondrial translation inhibitors (like tidazolamide).

#### 4.2.4 NAD+/NADPH

AML cells depend on DNA damage repair networks to withstand therapeutic stress and maintain a proliferative edge due to their distinctive immunometabolic pathways. The key reducing equivalents for ribonucleotide reductase (RNR), which catalyzes the conversion of nucleoside diphosphates (NDPs) to deoxyribonucleoside diphosphates (dNDPs) in order to construct the deoxyribonucleoside triphosphate (dNTP) pool required for DNA replication, are specifically provided by reduced nicotinamide adenine dinucleotide phosphate (NADPH) (120).

Nicotinamide phosphoribosyltransferase, or nicotinamide phosphoribosyltransferase (NAMPT), is the primary rate-limiting enzyme in the NAD+ remediation production pathway, which is heavily dependent on AML cells. Notably, aberrant NADPH accumulation is a result of mutations in the ME2 or IDH1 genes, and this enhances the malignant growth of AML cells.

NAMPT is a crucial rate-limiting enzyme in the NAD+ remediation pathway, which is extremely important to AML cells. When NAMPT is inhibited, the intracellular NAD+ pool is depleted, which sets off a chain of events that includes lipid peroxidation, oxidative phosphorylation impairment, decreased ATP synthesis, ROS buildup, and mitochondrial membrane potential disintegration. It should be noted that the SIRT family deacetylases’ activity is reliant on NAD+ levels and that its suppression results in aberrant histone acetylation, which impacts the control of genes linked to DNA repair, including BRCA1/2.

As an essential cofactor in PARP1/2-catalyzed processes, NAD+ plays a crucial regulatory function during DNA synthesis and repair. AML cells are much more sensitive to PARP inhibitors when NAD+ depletion is present because it not only prevents PARP-mediated repair of DNA single-strand breaks but also causes homologous recombination defective (HRD) phenotypes by down-regulating SIRT6’s deacetylation activity, which results in hyperacetylation and transcriptional repression of the BRCA1 promoter region (121).

Furthermore, by deacetylating important metabolic enzymes like PDH and IDH2, mitochondria-localized SIRT3 improves the effectiveness of oxidative phosphorylation, a mechanism that aids AML cells’ survival in a low-glycemic milieu. Clinical observations showed that chemoresistance is intimately linked to SIRT3 overexpression and that its particular inhibitor 17f causes mitochondrial malfunction and PARP1 hyperactivation, which in turn leads to cell death resembling parthanatos. But through the Preiss-Handler pathway, bacterial metabolites (like niacin) in the TME can restore the NAD+ pool. This bypass mechanism dramatically reduces the therapeutic effectiveness of NAMPT inhibitors (113). As a result, one crucial molecular indicator for predicting treatment resistance in AML cells is the degree of NAPRT expression.

Notably, the mitochondrial malic enzyme (ME2) catalyzes the decarboxylation of malate to produce pyruvate and regenerate NAD(P)H. This not only sustains nucleotide synthesis and TCA cycling flux, but it also inhibits 2-HG by producing α-ketoglutarate (α-KG), which in turn controls the activity of the TET enzyme and influences DNA hydroxymethylation and leukemic stem cell differentiation (122).

Through increased expression of SLC7A11, AML cells take over cystine in the immune system and prevent T-cell glutathione synthesis, which reduces the anti-tumor immune response. Immune checkpoint molecules like PD-L1 and CTLA4 are expressed more when the CREB3L2 transcription factor is activated by disrupted NAD+ metabolism. Notably, NK cell activity and NAD+ are intimately linked. *In vitro*-expanded iADAPT NK cells showed increased glycolysis and oxidative phosphorylation, and their NAD+/NADH ratio was markedly higher, which helped to maintain sustained cytotoxicity (123).

Because they operate together to regulate DNA repair, redox homeostasis, and immune metabolism, NAD+ and NADPH form a crucial metabolic network for the malignant evolution of AML. The current treatment bottleneck may be overcome by targeted intervention in their synthesis pathways or important regulatory nodes in conjunction with immunomodulators and medications that damage DNA. Future research should concentrate on creating NAD+ biosensors so that metabolic changes in AML cells may be dynamically monitored. Combining NAMPT inhibitors with CD73 blockers is a particularly intriguing approach since it reverses the immunosuppressive effects of adenosine while also blocking the niacin repair route. In-depth examination of the spatiotemporal heterogeneity features of metabolite gradients and DNA repair status in the TME of AML will be possible with the aid of spatial multi-omics technology. This will help to clarify the mechanism of microenvironmental metabolic collaboration and offer fresh suggestions for the improvement of customized treatment plans (**Table 1**).

**Table 1:** Metabolic profiles of immune cell subsets in AML.

Immune cell	Specific subtypes	Post-activation metabolic pattern				Metabolites/Enzymes	Mechanisms/pathway	Drugs/treatments	Research phase	Bibliography
		glycolysis	OXPHOS	Fatty acid oxidation	Glutamine metabolism					
T-cell	Primitive T-cell	↓	↑	↑↑	↓	glucose	Aerobic oxidative metabolism			(50)
	Regulatory T-cell	↓	↑	↑↑	↓	CPT1A, ACSL	Fatty acid metabolism pathways	Targeting CPT1A/ACS L inhibitors	preclinical	(149)
	Th1 cell	↑↑	↓	↓	↑	LDHA, GLUT1	PI3K-Akt-mTOR	2-DG(glucose inhibitor)	Phase II	(128)
	Th17 cell	↑↑	↑	↓	↓	Mitochondrial autophagy-related factors	HIF-1α/STAT3 pathway	Chloroquine (mitochondrial autophagy inhibitor)	Phase II	(149)
	Memory T -cell	↑↑	↑	↑↑	↓	Fatty acid	FAO/Mitochondrial Metabolism	Supplementary SCFA	Preclinical	(103)
	Toxic T-cell	↑↑	↓	↓	↑	Lactic acid, pyruvic acid	NFAT/IFN-γ pathway	MCT1/4 inhibitor (AZD3965)	Phase I	(42)
	DepletionCD8+T cell	↓↓	↑			Tox、PD-1	MCT1/4 inhibitor (AZD3965)	PD-1 inhibitor (navulizumab)	Approved (combined chemotherapy)	(86)
Macrophages	M1 Macrophages	↑↑	↓	↓	↓	Lactate、IFN-γ	GPR132/GPR65 signal	IFN-γ activator	Approved (topical application)	(8, 86)
	M2Macrophages	↓	↑↑	↑↑	↓	Polyamine, L-proline	TCA cycle/ETC complex	Metformin、Statins	Phase III (combined chemotherapy)	(150)
	Tumor-relatedMacrophages(TAM)	↑	Lactate metabolism (M2 polarization)	↑↑		Polyamine, L-proline	GPR132/GPR65 receptor signaling	Lactate transport inhibitor (MCT1/4 antagonist)	Phase I/II	(26)
neutrophil		↑↑			↑↑	Lactate	low mitochondrial dependence			(50)
NK	Resting NK		↑			Fatty acid、glucose	Basic mitochondrial respiratory chain			(151)
	Activated NK	↑	↑			LDHA、GLUT1、Lactate	PI3K-Akt-mTOR、GSK3β	IL-15/ALT-803 (enhanced glucose)	Phase II	(130)
	CD56bright NK (regulated)	↑↑	↑↑			Pyruvate, ROS	JAK-STAT signal	Lactate Scavenger (Bicarbonate Buffer)	preclinical	(36)
	CD56dim NK (toxic type)	↑↑	↑			Perforin, granzyme	ERK/AMPK pathway	Venetoclax (enhanced mitochondrial metabolism)	Approved (AML indications)	(112)
Dendritic cell	Tolerance DC	↑				Lactate	Autotolerance signaling pathway	Lactate scavenger (bicarbonate buffer)	preclinical	(36)
	Activate DC	↑		↓		Lactate	IFN-α production pathway	Epacadostat (IDO inhibitor)	Phase II(combined PD-1)	(6)
	pDC (plasma DC)	↑↑				Lactate	TLR7/9 pathway	Lactate dehydrogenase (LDH) inhibitor (GSK2837808A)	preclinical	(152)
MDSC (myelodysplasia)		Fatty acid oxidation (FAO)	glucose/OXPHOS mix			ARG1、ROS	STAT3/NO pathway	Metformin (inhibits OXPHOS)	Phase II (combined chemotherapy)	(153)

## 5 Impact of metabolic reprogramming on immunotherapy for acute myeloid leukemia

The fierce metabolic rivalry between leukemia cells and immune cells, which not only defines the immunosuppressive milieu but also directly influences the selection of subsequent therapeutic methods, is the primary cause of the therapeutic dilemma associated with AML. Targeted therapy and medication combinations have advanced significantly, and metabolic management has emerged as a major breakthrough in the treatment of AML. It’s important to remember that popular treatments like CAR-T cell therapy have clear drawbacks. The metabolic plasticity of distinct T cell subtypes varies greatly; memory T cells (TSCM) can sustain long-lasting anti-tumor activity through oxidative phosphorylation (OXPHOS), while Teff depend on the glycolysis pathway to quickly kill tumors but are prone to depletion. The balance of immunological reestablishment following transplantation determines the effectiveness of allogeneic HSCT, and accurate metabolic control can greatly increase the therapeutic effect’s longevity and lower the risk of complications.

### 5.1 Research on the regulation mechanism of targeted immune metabolism

In AML cells, targeting metabolic enzymes like ME2 (e.g., Na_2_EA) lowers ATP levels and triggers apoptosis. Chemotherapy’s effect is greatly increased when the ATP action is blocked by blocking the P2X7 receptor. While agonists increased apoptosis and bolstered anti-tumor immune responses, P2X7 receptor antagonists were discovered to efficiently suppress AML cell proliferation and metastasis (115). Regarding metabolic intervention, the arginine deprivation agent ADI-PEG20 specifically kills leukemia cells (124); the ASCT2 inhibitor V-9302 selectively kills AML cells by blocking glutamine uptake while maintaining the metabolic requirements of T cells (125); and APO866 inhibits PARP activity by depleting NAD+ and, in concert with Olaparib, causes HR-deficient AML cell death.

While BPTES and CB-839 decreased glutamine uptake by blocking GLUT function, the GLS inhibitor CB-839 markedly slowed the growth of leukemia (126). By preventing GS activity, SETD2-IN-1 inhibited glutamine synthesis. OXPHOS is inhibited by statins, a cholesterol synthesis inhibitor, and metformin, a mitochondrial complex I inhibitor (124). Research has shown that both increase T and NK cell function while blocking MDSCs function, strengthening the antitumor immune response. By preventing mitochondrial oxygen consumption, hydroxyurea indirectly inhibits OXPHOS, whereas pim kinase inhibitors stop leukemia cell growth as well as important protein kinase activities like AMPK (70).

Regarding targeted treatments, Ivosidenib and Enasidenib reduce 2-HG levels by inhibiting IDH mutase activity, hence reversing epigenetic aberrations (36). With the disclaimer that differentiation syndromes may develop in roughly 10% of patients, phase III therapeutic trials have demonstrated overall remission rates of 30–40% for monotherapy in relapsed/refractory AML (127). Fatty acid metabolism-focused intervention techniques have demonstrated that inhibiting transporter proteins like CD36 lowers the consumption of exogenous fatty acids, while targeting important FAO enzymes like CPT1A or ACSL damages the cellular energy supply of AML (128). Targeting both FAO and synthetic pathways (for example, by blocking FASN or ACC) greatly increases efficacy and lowers the likelihood of drug resistance (103).

Lactate produced by STAT5 can be utilized as a prediction marker for PD-(L) 1 immunotherapy in terms of immunometabolic control. It was discovered that PD-1/PD-L1 inhibitors were effective in treating patients with AML that was increased by STAT5-mediated glycolysis (58). By preventing lactate export and removing the inhibition of immune cells by lactate, the dual MCT1/4 inhibitor AZD3965 caused intracellular acidosis (42). T-cell depletion is reversed by metabolic reprogramming; for example, supplementing with pyruvate or α-KG increases T-cell function and restores TCA cycling activity (129).

Regarding immunological regulatory systems, the SHP-1-MYC axis governs CD47-dependent macrophage escape, and suppressing SHP-1 increases MYC degradation and suppresses CD47 expression to improve phagocytosis (83). TIM3 and PD-1 are important immunological checkpoints for NK cell failure, and CAR-TIM3 NK-92 cell treatment can successfully reduce NK cell depletion (61). In addition to boosting the immunological response of T cells, PD-1/PD-L1 blockers cause MDS/AML primitive cells to undergo apoptosis.

Through JAK-STAT signaling, IL-15/ALT-803 pretreatment increases HK2 expression, improves glycolytic flux, and increases the ability of NK cells to infiltrate tumors and destroy them (130).

By stimulating NK cells’ mitochondrial metabolism and boosting ATP production, Venetoclax dramatically increased anti-tumor efficacy (126). By lowering SAM levels, methionine restriction restores T cell depletion and suppresses PD-L1 expression. Treg cell reliance on BCAAs was diminished and OXPHOS activity was inhibited by blocking the SLC7A5 transporter protein (131). The AMPK activator AICAR reduced the inflammatory response by preventing M1-like macrophages from secreting TNF-α. Notably, Venetoclax increased mitochondrial respiration and OXPHOS through IKKβ/NF-κB signaling, which greatly increased the killing effectiveness of NK cells (70). When combined with daltuzumab, CD38 knockdown prevented Fc receptor-mediated self-killing while preserving NK cell KIR diversity and improving AML detection.

According to recent research, Olutasidenib, an IDH inhibitor, may improve the effectiveness of PD-1 inhibitors by altering the immune milieu by lowering 2-HG (129). When used with Magrolimab, Ivosidenib markedly increased macrophage phagocytosis. By enhancing T-cell infiltration, LDHA inhibitors worked in concert with IDH inhibitors (58). Through AMPK activation and NF-κB p65 phosphorylation downregulation, metformin therapy decreased immunosuppressive cytokine production (84).

### 5.2 Combined treatment strategies

Glycolysis inhibition and OXPHOS activation in bimodal synergistic therapy: the combination of 2-DG (a glycolysis inhibitor) and metformin (an OXPHOS enhancer) causes the metabolic collapse of AML cells while improving T cell mitochondrial metabolic performance (111). By blocking ME2 to lower NADPH levels and adding BSO (GSH synthesis inhibitor) to enhance the ROS effect in concert, the oxidative defense barrier of AML cells was successfully broken (113, 157). Cytarabine with a cysteine thiol-based designed polymer vaccine (PDS platform) greatly improve the effectiveness of antigen presentation and trigger particular T-cell anti-leukemia responses (132). GPX4 inhibitors (e.g., salazosulfapyridine) greatly increased the immunogenic cell killing consequences of ROS buildup caused by cysteine depletion or suppression of GSH formation (119). Lactate-mediated dual immunosuppression was effectively disarmed by LDHA inhibitors (e.g., GSK2837808A) in conjunction with anti-PD-1 antibodies through a metabolic checkpoint blockage mechanism (5).

Combining demethylating medications with Venetoclax overcomes apoptosis resistance and eliminates leukemic stem cells (133); combining with FLT3 inhibitors targets co-mutagenic signaling pathways (82); and combining with demethylating medications can restore DNA methylation homeostasis, as demonstrated by clinical studies. Several phase II clinical trials are presently being conducted. The triple drug regimen of FLT3 inhibitor, glycolysis inhibitor (2-DG), and anti-PD-L1 antibody can synchronously block the energy supply and the immune checkpoint pathway through the metabolic-immune dual-targeting mechanism (129). IDO1 inhibitor (Epacadostat) and PD-L1 blockade can also relieve the dual immunosuppression caused by tryptophan depletion and checkpoints, restoring T-cell clonal expansion (133). The ERK1/2 pathway is activated while P70S6K phosphorylation is decreased and 4EBP1 is partially dephosphorylated by the mTORC1 inhibitor (rapamycin or quercetin). When U0126 and ERK1/2 are combined, 4EBP1 and P70S6K dephosphorylation are improved, and cytotoxicity is markedly increased. Through a synergistic inhibition of protein synthesis, this combined regimen accelerated cell death (63). Even greater 4EBP1 dephosphorylation effects and cytotoxicity were obtained by further combination with the AKT inhibitor MK2206, which was noticeably better than single-passage inhibition (67). The delivery of NAD+ precursors, such as nicotinic acid, to AML cells by bone marrow stromal cells through gap junctions reduces the effectiveness of NAMPT inhibitors, indicating the necessity of investigating combination treatment regimens using NAPRT inhibitors, such as 2-hydroxynicotinic acid. Specific tyrosine kinase inhibitors (TKIs) targeting FLT3 kinase are the core strategy for treating FLT3-ITD AML. Although TKIs (such as quizartinib and midoshuin) can temporarily relieve symptoms, they are prone to drug resistance (SPHK1/S1P-β-catenin compensatory activation) (124). It is important to consider how TPI-1 affects normal immune cell activity, even though it sensitizes to chemotherapy through a dual metabolic-immune mechanism (134). Future precision intervention can be achieved by the development of single-cell kinomics technology to address the treatment resistance brought on by the heterogeneity of kinase activity in subpopulations of LSCs. In order to improve efficacy, synergistic inhibitors of various pathways should be developed because targeting a single important metabolic node (PFKP, GLS1, etc.) may cause compensatory pathways to be activated.

### 5.3 Systemic risk of metabolic inhibitors

Targeted metabolism has opened up new avenues for the treatment of AML, but it faces challenges in clinical translation. The core issue is that targeting metabolic pathways (such as glycolysis and OXPHOS) is also crucial for the physiological functions of normal cells, making systemic metabolic inhibitors a double-edged sword and prone to causing widespread “off-target” toxicity (135). For instance, although the mitochondrial complex I inhibitor IACS-010759 demonstrated strong anti-leukemia activity in preclinical trials and entered clinical trials, the trials were eventually terminated due to dose-limiting toxicities (DLTS) such as peripheral neuropathy and lactic acidosis.

Given the inherent risks of systemic drug administration, the development of AML cell-selective drug delivery strategies has become an inevitable requirement. The core is to increase the drug concentration at the tumor site and reduce the systemic circulating concentration to protect normal tissues. Nanocarrier technologies (such as liposomes, polymer nanoparticles, and gold nanoparticles) can encapsulate metabolic inhibitors and enhance tumor targeting. Antibody-drug conjugates (ADCs) combine the targeting power of monoclonal antibodies with the cytotoxicity of small molecule drugs. They link glycolytic or OXPHOS inhibitors to CD33/CD123 targeted antibodies through linkers. After endocytosis, the drugs are released to precisely destroy AML cells, while normal cells are spared due to their low expression of target antigens (136).

However, the development of targeted delivery systems still faces challenges, including optimizing drug delivery efficiency and drug release kinetics, reducing immunogenicity, and controlling production costs (137). In the future, the combined application of targeted metabolic inhibitors and immunotherapies (such as CAR-T, bispecific antibodies, and ICI) holds great potential. It can not only directly kill AML cells but also improve the metabolic inhibition state of the TME, enhance the anti-tumor immune response, and achieve synergistic effects (**Table 2**).

**Table 2:** Targeted therapy, metabolic regulation and combined treatment strategies for AML.

Form	Targets/mechanisms	Drugs/intervention methods	Mechanism of action	Effect	Combined therapy	Bibliography
targeted therapies	Reversal of metabolic inhibition (TCA cycle)	Exogenous pyruvate/alpha-ketoglutarate (α-KG)	Supplementation of metabolic intermediates restores TTCA activity and reverses depletion phenotype	Release of T-function inhibition		(129)
	ME2 metabolizing enzyme	Na_2_EA	Reduces AML ATP levels and induces its death	Specific Targeted Leukemia		(113)
	P2X7 receptor	P2X7 antagonist/agonist	Antagonists inhibit AML growth metastasis, agonists promote apoptosis and enhance anti-tumor immunity	Enhances the effect of chemotherapy, but needs to balance agonism and antagonism		(115)
	ASCT2 (glutamine transporter)	V-9302	Blocking glutamine uptake and selective starvation of AML	Retention of T glutamine metabolic requirement		(125)
	Arginine metabolism	ADI-PEG20	Arginine deprivation agent that selectively kills leukemia	Targeting arginine-dependent tumors		(154)
	MCT1/4 (Lactate transporter)	AZD3965	Blocking Lactate efflux, inducing death by intra-AML acidosis, and relieving immunosuppression by Lactate	Directly kills AML and restores immune activity		(42)
	CD47-SHP-1-MYC Axle	SHP-1 inhibitor	Inhibition of SHP-1 leads to MYC degradation, deregulation of CD47 expression inhibition, and enhanced phagocytosis of Macrophages	Blocking immune escape mechanisms in AML		(83)
	FAO(CPT1A/ACSL)	CPT1A/ACSL inhibitor	Weakening of AMLFatty acid oxidative energy supply	Need to combine other metabolic targets (e.g., FASN or ACC) to enhance efficacy	Dual targeting of FAO and synthetic pathways (e.g., inhibition of FASN+CPT1A) reduces drug resistance	(103)
	CD36 (Fatty acid uptake)	CD36 inhibitor	Blocking exogenous fatty acid utilization	Limiting AML energy sources	Combined FAO Inhibitor Enhancement	(103)
	NK immune checkpoint (TIM3/PD-1)	CAR-TIM3 NK-92 therapy	Mitigation of NK depletion	Enhancement of NK anti-tumor activity		(61)
	PD-1/PD-L1	PD-1/PD-L1 blockers	Blocking PD-1/PD-L1 binding enhances T-immune responses and induces AML apoptosis	Effective in patients with STAT5-induced glucose/Lactate accumulation; Lactate can be used as a biomarker	In combination with glucose inhibitors (e.g., LDHA inhibitors)	(58)
metabolic regulation	JAK-STAT signal/HK2	IL-15/ALT-803 pretreatment	Up-regulation of HK2 expression enhances NK glucose fluxes	Enhancement of NK tumor infiltration and killing capacity		(130)
	mitochondrial metabolism	Venetoclax	Activates NK mitochondrial metabolism and increases ATP production	Enhanced anti-tumor activity		(126)
	Glutamine metabolism (GLS/GLUT/GS)	CB-839/BPTES/SETD2-IN-1	Inhibits glutamine transport or synthesis	Inhibition of leukemia progression		
	Methionine metabolism	Methionine restriction	Reduced SAM levels and inhibited PD-L1 expression	Reversing T depletion		(116)
	BCAAs metabolism/SLC7A5	SLC7A5 inhibitor	Decreased OXPHOS activity and reduced Treg dependence	Dual regulation of AML and the immune microenvironment		(131)
	OXPHOS	Metformin/Statins/Hydroxyurea	Inhibition of mitochondrial complex I/cholesterol synthesis/oxygen consumption, enhancement of T/NK function, inhibition of MDSCs	Improved anti-tumor immune response		(155, 94, 150)
	AMPK inflammation regulation	Acadesine(AICAR)	Inhibition of TNFα secretion from M1Macrophages reduces inflammation	Need for precise stratification (e.g., metabolic differences in AML subtypes)		(70)
	Pim kinase	Pim kinase inhibitor	Inhibition of leukemic growth and proliferation and kinases such as AMPK	Potential multi-target effects		(70)
	Microenvironmental Lactate removal	LDH nanoparticles/bicarbonate buffer	Degradation of Lactate or neutralization of the acidic microenvironment to restore T function	Improvement of the immunosuppressive microenvironment		(36)
Combined therapy	glucose+OXPHOS dual targeting	2-DG + Metformin	glucose inhibits combined OXPHOS activation, forcing AML metabolism to collapse and activating T cells	Metabolic-immune synergy	bimodal therapy	(1)
	Oxidative Stress Breakthrough	ME2 inhibitor + BSO	Reduces NADPH and inhibits GSH synthesis, amplifies ROS	Breaking through the AML oxidative defense barrier	Synergistic enhancement of oxidative damage	(113)
	Cysteine-targeted immunization	PDS platform vaccine + cytarabine	induction of cysteine thiol-dependent antigen presentation and activation of T cells	Enhancement of chemotherapy immunogenicity	Combining chemotherapy with immune activation	(132)
	Oxidative damage + iron death	Cysteine depletion + GPX4 inhibitor	Synergistic Killing of AML by ROS Accumulation and Iron Death	Need for balanced immune protection	Double oxidative stress induced	(119)
	Lactate metabolism + immune checkpoints	LDHA inhibitor + anti-PD-1 antibody	Resolving Lactate-mediated immunosuppression and blocking PD-1	For patients with high Lactate microenvironments	Metabolic-immune combined	(43)
	NAD+ metabolism/PARP	APO866 + Olaparib	Depletion of NAD+ inhibits PARP and synergistically induces HR-deficient AML death	AML for DNA repair defects	Synergistic use of PARP inhibitors	(124)
	Immunometabolic double suppression	IDO1 inhibitor + PD-L1 blockade	Release of tryptophan depletion and checkpoint inhibition and restoration of T amplification	Dual immune activation	Epacadostat + PD-L1 antibodies	(133)
	Synergistic inhibition of signaling pathways	BEZ235 + R848	Inhibition of the PI3K/Akt/mTOR signaling pathway to disrupt tumor escape mechanisms	Disrupting Tumor Immune Escape	Combined application of PI3K/mTOR inhibitors and TLR7/8 agonists	(64)
	Microenvironmental NAD+ compensation	2-Hydroxynicotinic acidcombined program	Blockade of bone marrow stromal delivery of NAD+ via niacin	Overcoming microenvironmental drug resistance	NAMPT inhibitor + NAPRT inhibitor	(124)
	Kinase-metabolism synergy	FLT3 inhibitor + 2-DG + anti-PD-L1 antibody	Synchronized blockade of energy supply and immune checkpoints	Targeting FLT3 mutant AML	Metabolic-immune dual targeting	(156)
	IDH mutation	Ivosidenib/Enasidenib	Inhibition of mutase activity reduces 2-HG levels and reverses epigenetic abnormalities	Single agent remission rate 30-40%, may trigger differentiation syndrome (~10%)	Combined demethylating drug (55% complete remission rate); combined with Venetoclax to overcome resistance	(129)
	Epigenetic + Apoptosis Targeting	IDH inhibitor + Venetoclax	Reversal of epigenetic abnormalities and induction of BCL-2-dependent apoptosis		Overcoming leukemia stem cell resistance	(75)

### 5.4 Progress in research on metabolic potentiation therapy

#### 5.4.1 CAR-T

The foundation of CAR-T therapy, which ushers in a new age of cancer treatment, is the genetic modification of the patient’s T-cells to enable them to recognize and effectively destroy cancer cells. This novel treatment works against tumors in three ways: first, the altered T cells can directly release cytotoxins to kill tumor cells; second, the cytokines they release can stimulate additional immune cells to fight together; and third, the immunomodulatory factors they release can significantly increase the T cells’ ability to proliferate and function (138).

The immune metabolic process is intimately linked to the clinical effectiveness of CAR-T treatment as a paradigm of T cell engineering application. While Teff, which use glycolysis as their primary metabolic mode, are prone to functional exhaustion even though they can have quick cytotoxic effects, memory T cells (TSCM) primarily get their energy from the oxidative phosphorylation (OXPHOS) pathway, which has long-term survival advantages and sustained anti-tumor activity. Notably, the metabolic properties of T cells are directly determined by their differentiation state (initial, memory, effector), and this relationship has a significant impact on the longevity and therapeutic effects of CAR-T cells (139).

Two major obstacles to treating AML with CAR-T therapy are TME inhibition and T cell depletion. T cell activity and proliferative potential are markedly inhibited by lactic acid accumulation in the microenvironment (for more information, see the previous section). Furthermore, AML cells overexpress cholesterol synthase, which reduces the amount of cholesterol in the milieu and hinders CAR-T cell signaling and membrane fluidity (140). Then, by disrupting their metabolic pathways, tumor metabolites including kynurenine (Kyn) and ketoglutaric acid prevent T cells from functioning. Moreover, AML cells accomplish immunological escape by a variety of means, including release of inhibitory cytokines (IL-10, TGF-β), activation of immune checkpoint molecules (PD-L1, TIM3), and downregulation of antigen expression (e.g., HLA deletion).

The following regulatory approaches are recommended in order to alleviate cholesterol deficiency in the AML microenvironment: HMG-CoA reductase overexpression to restore CAR-T cell membrane function (141). introduction of the anti-ketoglutarate lyase gene into the TME, which neutralizes toxic metabolites like Kyn and breaks down inhibitory metabolites. When it comes to metabolic pathway intervention, 2-deoxy-D-glucose (2-DG) can be employed to stimulate the formation of memory T cells and inhibit glycolysis. The glutamine antagonist DON or L-arginine supplementation can increase OXPHOS activity and cause the stem cell-like memory T cells (TSCM) phenotype.

To decrease effector T-cell differentiation and increase CAR-T survival *in vivo*, it is advised to substitute IL-2 with IL-7 and IL-15 while optimizing cytokines and media. In contrast to human platelet lysate, which helps to enrich central memory T cells (TCM) subpopulations, myostatin decreases medium acidification (142). T-cell function inhibition was reversed by blocking kynurenine production with IDO inhibitors (serum Kyn levels were inversely linked with CAR-T effectiveness). The T-cell depletion status can be lifted by a combination of PD-L1 inhibitors.

Furthermore, combination treatments can successfully overcome microenvironmental restrictions. For example, combining CAR-T cells with immune checkpoint inhibitors, chemotherapy, venetoclax, or demethylating medications can greatly increase efficacy and overcome drug resistance (143).

T-cell metabolic profiles and metabolic suppression in the TME both influence the therapeutic efficiency of CAR-T cells at the same time. The durability and anti-tumor effectiveness of CAR-T cells can be greatly increased by altering metabolic pathways, improving culture conditions (e.g., by adding particular cytokines or metabolites), and combining metabolic medications or immunomodulators. In order to support advancements in CAR-T in the treatment of resistant cancers like AML, future research should concentrate on the mechanism of metabolic-immune interactions and create specific intervention strategies that target metabolic inhibition in the TME.

#### 5.4.2 Hematopoietic stem cell transplantation

The successful reconstitution of the immune system is the key to the effectiveness of bone marrow transplantation, a significant treatment for hematologic malignancies. Numerous factors, such as the patient’s age, the kind of transplant, the source of the donor, the use of T-cell scavengers, and graft-versus-host disease (GVHD), affect this intricate and changeable process (144). Remarkably, the majority of AML treatment plans weaken the immune system and lower leukemic immunogenicity; these side effects could be even worse by allogeneic hematopoietic stem cell transplantation (alloHSCT) (145).

There are three main stages to the immune reconstitution process: Donor-derived immune cells (such as T-cells, B-cells, natural killer cells, etc.) predominate in phase I (one to three months after transplantation), which is characterized by their rapid post-transplant expansion but lack of persistent immunological homeostasis. Stage 2 (three to six months after transplantation): functional heterogeneity persists but the immune cell composition tends to settle. While the number of Tregs increases and the proportion of initial T cells decreases, the proportion of T cells and B cells gradually recovers. This is most likely due to the immunosuppressive milieu that occurs after transplantation. The immunological profile and function were further recovered in stage three (6–12 months after transplantation), while some abnormalities persisted, such as a lowered initial T cell ratio and a continuously raised Tregs ratio. The long-term survival and quality of life of patients are directly impacted by the level of immune reconstitution perfection at this point (146).

There are two primary pathways that comprise the metabolic regulatory mechanisms of immunological reconstitution: extracellular and intracellular. By attaching to the appropriate ligands, inhibitory receptors (IRs) on effector cell surfaces in the intracellular route suppress anti-tumor immune responses. The anti-tumor activity of T cells is considerably inhibited, for instance, when the TIGIT ligand expressed on the surface of tumor cells binds to the TIGIT receptor on the surface of T cells. Clinical findings showed a strong correlation between an increased risk of relapse and higher levels of TIGIT expression on the surface of CD4 T cells in individuals with AML relapse. Likewise, tumor cells and immunosuppressive cells express the CD161 ligand, which binds to the CD161 receptor on T cells’ surface to limit T cell activity. Significantly higher PD-1 expression on the surface of CD8 T cells and raised CD161 expression on the surface of CD4 T cells are both observed in individuals with AML relapse; these alterations are associated with an increased risk of disease recurrence.Subsets of immunosuppressive cells are important in the extracellular route (147). The immunosuppressive milieu following bone marrow transplantation may be the reason for the decline in the fraction of initial Treg, despite an increase in the overall proportion of Tregs. T cell and NK cell activity can be efficiently suppressed by MDSCs, a subset of bone marrow-derived cells with immunosuppressive properties. Following bone marrow transplantation, MDSCs levels have been shown to be temporarily increased and typically revert to normal after a year.

The immune reconstitution process following bone marrow transplantation is greatly influenced by a number of non-genetic factors in addition to genetic ones. Among these, cytomegalovirus (CMV) infection causes remodeling of the NK cell profile in addition to changing the variety and makeup of the TCR pool. Furthermore, clonal proliferation of the T-cell pool can be induced by CMV infection, which impacts the functional characterisation of the TCR pool (147).

Graft-versus-host disease (GVHD), the most frequent side effect of allo-HCT, is mostly caused by donor T-cells attacking host tissues. Its typical clinical symptoms include intestine, liver, and skin damage along with systemic inflammatory reactions. From an immunometabolic standpoint, GVHD is characterized by excessive secretion of pro-inflammatory cytokines including IFN-γ and IL-17 as well as aberrant Th1 and Th17 cell activation. Interestingly, GVHD may also encourage the growth of immunosuppressive cell subpopulations, which would reduce the immune response against tumors. The primary therapeutic therapy options at the moment are donor lymphocyte infusion (DLI), immunosuppressive drugs, and anti-thymocyte globulin (ATG) (148). The immune reconstitution process may also be hampered by other pathogenic infections, such as Epstein-Barr virus (EBV), germs, etc., in addition to CMV.

Future research should concentrate on the main mechanisms of the role of immunomodulatory molecules such as TIGIT and CD161 in AML relapse in order to fully comprehend and maximize immunological reconstitution following bone marrow transplantation. These research will assist clarify the crucial role of immunometabolic regulation in long-term problems following bone marrow transplantation and will serve as a significant foundation for the development of therapies to target immunomodulation for AML relapse.

## 6 Conclusion

Novel ideas for treating AML have emerged as a result of the complex interactions between immune cell metabolism and AML. Targeting AML metabolic pathways, controlling nutrient metabolism, mending the BMM, and reviving immune activity have all been shown to be effective tactics. These approaches can work in concert with treatments like hematopoietic stem cell transplantation (HSCT) and CAR-T cell therapy. To improve the effectiveness of immunotherapy regimens, more clarification of the metabolic processes causing AML immune escape and the metabolic needs of immune cells is required. It is crucial to recognize that the metabolic reprogramming of AML cells affects immune cell metabolic reprogramming as well as antigen presentation and immunological detection. As a result, immune cells’ functional characteristics change, which in turn causes the AML immunological microenvironment to undergo dynamic modifications.
